# *In silico* Methods for Design of Kinase Inhibitors as Anticancer Drugs

**DOI:** 10.3389/fchem.2019.00873

**Published:** 2020-01-08

**Authors:** Zarko Gagic, Dusan Ruzic, Nemanja Djokovic, Teodora Djikic, Katarina Nikolic

**Affiliations:** ^1^Department of Pharmaceutical Chemistry, Faculty of Medicine, University of Banja Luka, Banja Luka, Bosnia and Herzegovina; ^2^Department of Pharmaceutical Chemistry, Faculty of Pharmacy, University of Belgrade, Belgrade, Serbia

**Keywords:** kinase inhibitors, rational drug design, molecular modeling, drug discovery, pharmacophore

## Abstract

Rational drug design implies usage of molecular modeling techniques such as pharmacophore modeling, molecular dynamics, virtual screening, and molecular docking to explain the activity of biomolecules, define molecular determinants for interaction with the drug target, and design more efficient drug candidates. Kinases play an essential role in cell function and therefore are extensively studied targets in drug design and discovery. Kinase inhibitors are clinically very important and widely used antineoplastic drugs. In this review, computational methods used in rational drug design of kinase inhibitors are discussed and compared, considering some representative case studies.

## Kinases As Targets For Developing Anticancer Drugs

Kinases belong to a large family of enzymes that catalyze transfer of high energy phosphate group from adenosine triphosphate (ATP) to substrates, such as proteins (the protein-tyrosine kinases, the serine-threonine specific kinases), lipids (phosphatidylinositol kinases, sphingosine kinases), carbohydrates, and nucleic acids (Duong-Ly and Peterson, 2013). Phosphorylation of the substrate modulates its activity and/or interaction with other molecules leading to different physiological responses. It is estimated that 50% of all proteins are constantly undergoing reversible phosphorylation and dephosphorylation, which emphasizes the role of protein kinases in almost all aspects of cell function, including proliferation, cell growth, apoptosis, and signal transduction (Graves and Krebs, 1999; Manning et al., 2002).

Dysregulated, overexpressed, or mutated protein kinases are found in many diseases, including cancer, and over the past two decades they became extensively examined targets for the development of new antineoplastic drugs (Blume-Jensen and Hunter, 2001; Cohen, 2002). There are 53 kinase inhibitors (KIs) currently approved by the FDA (FDA, 2019), while over 200 potential inhibitors are in different phases of clinical trials worldwide (Carles et al., 2018). Majority of the approved drugs are orally active and effective against various malignancies (Table 1; Roskoski, 2019a,b).

**Table 1:** Therapeutic indications of selected FDA-approved protein kinase inhibitors.

**Therapeutic indication**	**Drug**
Breast cancer	Everolimus, lapatinib, neratinib, palbociclib, ribociclib
Non-small cell lung cancer	Afatinib, alectinib, brigatinib, ceritinib, crizotinib, dabrafenib, dacomitinib, erlotinib, gefitinib, lorlatinib, osimertinib
Leukemia	Bosutinib, dasatinib, gilteritinib, ibrutinib, imatinib, midostaurin, nilotinib, ponatinib
Melanoma	Binimetinib, cobimetinib, dabrafenib, encorafenib, trametinib, vemurafenib
Thyroid cancer	Cabozantinib, lenvatinib, vandetanib
Renal cancer	Axitinib, pazopanib, sorafenib, temsirolimus
Gastrointestinal cancer	Regorafenib, sunitinib

**Figure 1:**
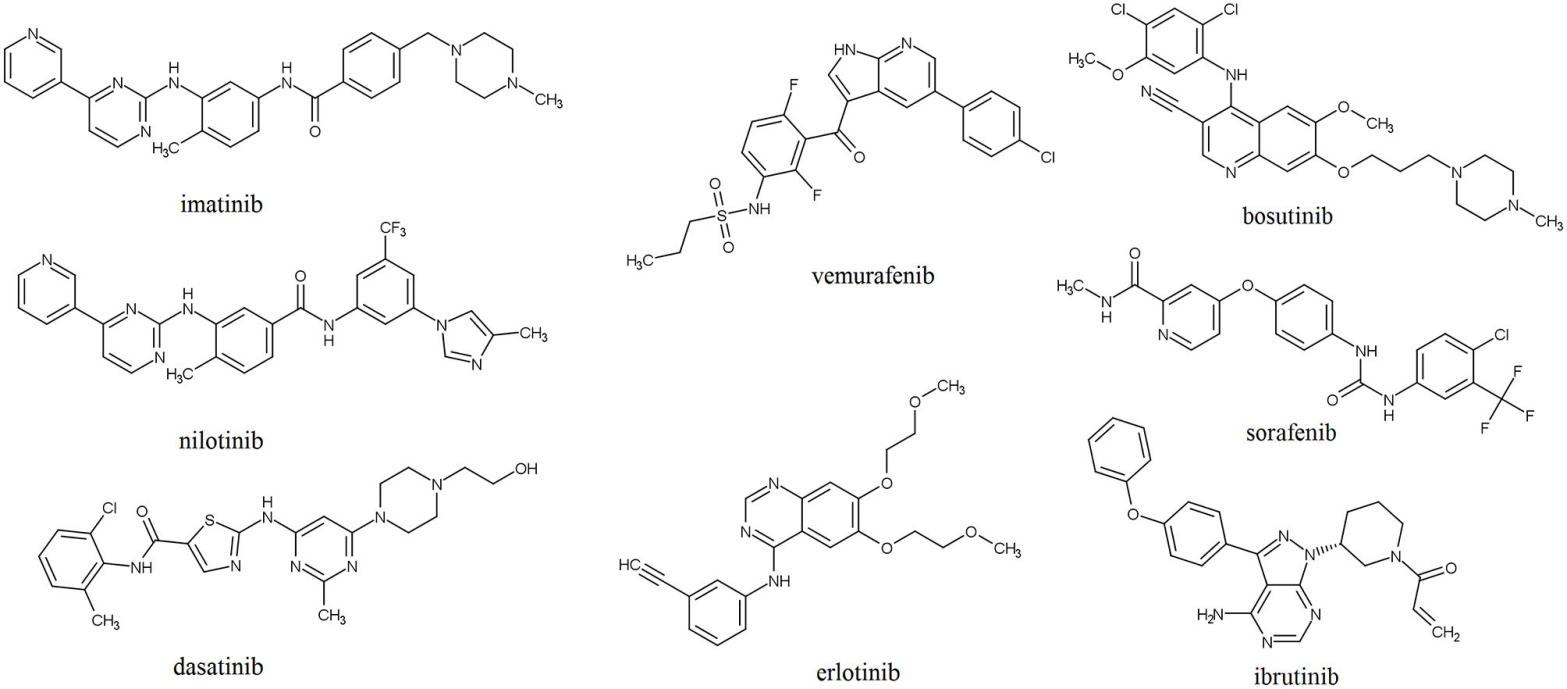
Structures of selected protein kinase inhibitors that have been approved for clinical use.

Structures of the selected KIs commonly used for treatment of cancer are shown in Figure 1. These drugs target different protein kinases that are frequently upregulated in cancer cells. The epidermal growth factor receptor (EGFR) is a member of the ErbB family of tyrosine kinase receptors that is overexpressed or mutated in non-small cell lung cancer and represents the primary target for drugs such erlotinib and gefitinib (Bethune et al., 2010). Lapatinib and neratinib bind to intracellular domain of the human epidermal growth factor receptor 2 (HER2/neu), another member of the ErbB tyrosine kinases, which elevated levels are found in approximately 20–30% of breast cancers (Collins et al., 2019). Imatinib possesses activity against non-receptor breakpoint cluster region (Bcr)-Abelson leukemia virus (Abl) tyrosine kinase that is formed as a result of a chromosome rearrangement and has been implicated in the pathogenesis of nearly all cases of chronic myeloid leukemia (CML) and acute lymphoblastic leukemia with the Philadelphia chromosome (Iqbal and Iqbal, 2014). Although imatinib is a relatively specific Bcr-Abl inhibitor, it also inhibits the CD117 tyrosine kinase associated with gastrointestinal stromal tumors and has consequently been approved for this indication (Buchdunger et al., 2000). The vascular endothelial growth factor family of receptors (VEGFR) contains a tyrosine kinase domain which activation can lead to induction of signaling pathways that regulate cell proliferation, survival, and promotion of tumor angiogenesis (Morabito et al., 2006). Agents that target VEGFR, including lenvatinib, sorafenib and vandetanib, are frequently used for treatment of thyroid cancers. Vemurafenib, dabrafenib, and encorafenib target BRAF, a serine/threonine protein kinase which mutation is expressed at about 50–60% of cutaneous melanomas where it leads to continuous activation of mitogen-activated protein kinase (MAPK) pathway and uncontrolled proliferation of cancer cells (Yu et al., 2019).

### Structure of Protein Kinases

**Figure 2:**
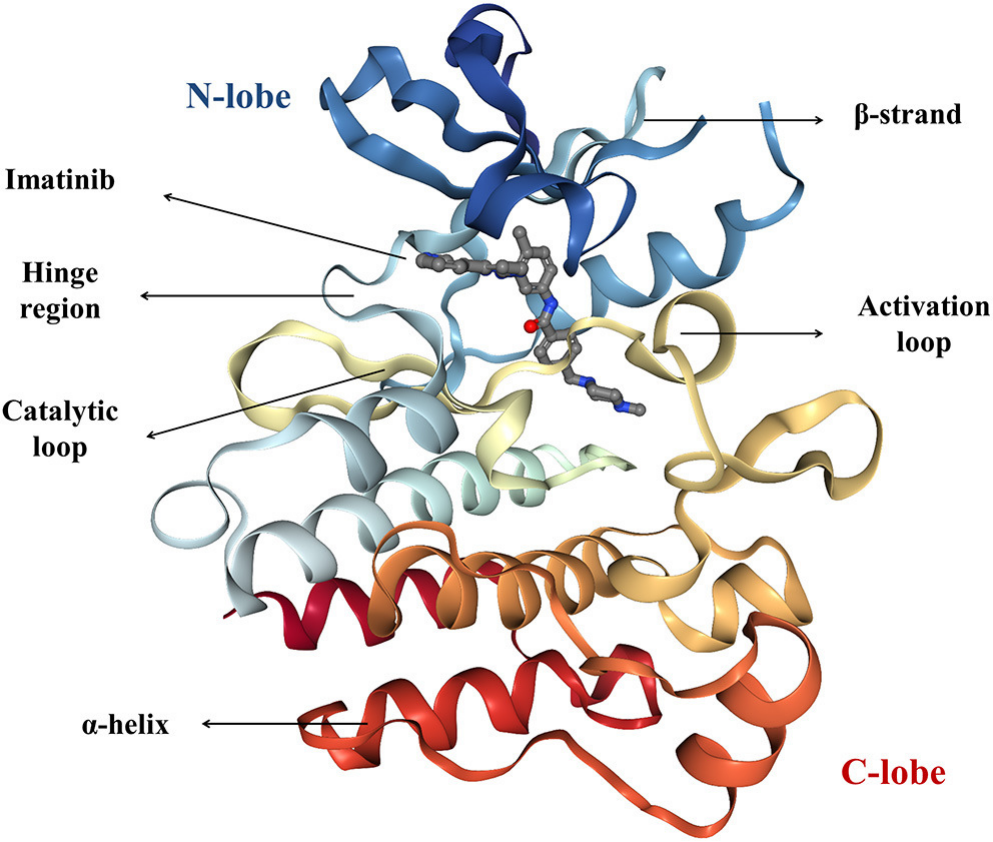
The crystal structure of imatinib-bound form of the Abl kinase (PDB accession code: 2HYY), colored as rainbow from N-lobe (blue) to C-lobe (red). Imatinib is represented as ball and stick.

The human genome encodes at least 518 protein kinases (Manning et al., 2002). Out of them, 478 share highly conserved catalytic domains. The remaining 40 do not share the sequence similarity, but their folding is similar to the folding of “typical” PKs (Caballero and Alzate-Morales, 2012). In 1991, Knighton solved the X-ray structure of cyclic AMP-dependent PK and described its structure for the first time. This description can apply to all currently known protein kinases. The characteristic architecture of the catalytic domain of PK consists of a small, amino-terminal N-lobe and a large α-helical carboxy-terminal C-lobe which are connected with a small hinge region (Figure 2; Knighton et al., 1991). The N-terminal lobe is dominated by five β-strands (β1–β5) and one conserved α-helix (helix C) that occurs in active (αC-in) or inactive (αC-out) orientations. The C-lobe consists of eight α-helices and four short conserved β-strands (β6–β9) which include residues that participate in the phosphorylation of protein substrates. The small and large lobes form a catalytic cleft where ATP binds (Knighton et al., 1991; Roskoski, 2019a). The hydrophobic residues of the cleft form a binding pocket for ATP. The charged residues in the active site bind and position the γ-phosphate of ATP and divalent cation and take part in the catalysis (Knight et al., 2007).

Conserved residues play crucial roles in positioning ATP, stabilizing the active-conformation and in the catalytic mechanism, and they are mostly found in and around the active site but also in other parts of the protein kinase domain (Knight et al., 2007). Almost all protein kinases possess a conserved K/E/D/D (Lys/Glu/Asp/Asp) signature that is important for the catalysis. Lysine and glutamic acid residues belong to the N-lobe, and the two aspartic acid residues are found in the C-lobe. Lysine residue binds to the α- and β-phosphates of ATP. Formation of the salt bridge between the carboxylate group of aspartic acid and the amino group of lysine stabilizes its interactions with the α- and β-phosphates, and it is required for kinase activation (Roskoski, 2015, 2019a,b).

The N-lobe contains a conserved flexible glycine-rich GxGxxG motif (also called P-loop) between β1 and β2 that folds over the nucleotide and places the γ-phosphate of ATP during the catalysis (Taylor and Kornev, 2011). As mentioned above, lysine from the β3-strand forms a salt bridge with the conserved glutamate near the center of the protein-kinase αC-helix which is necessary for the formation of the active enzyme, and this structure corresponds to the “αC-in” conformation (Roskoski, 2015, 2019a,b).

The C-lobe is important for both the protein-substrate binding as well as nucleotide binding (Roskoski, 2015). The C-lobe contains a mobile activation loop of 20–30 residues which can take open or closed conformation. The activation loop begins with the DFG motif (Asp-Phe-Gly) and extends up to an APE motif (Ala-Pro-Glu) (Modi and Dunbrack, 2019). In the active conformation a divalent metal ion, Mg^2+^ (or sometimes Mn^2+^), interacts with a highly conserved aspartic acid residue from the DFG motif. It coordinates with the α and γ phosphates of ATP and facilitates the phosphorylation and coordinates the ATP binding (Adams, 2001). At the other end, glutamic acid from APE motif is fixed by the formation of a salt bridge with arginine from the C-lobe (Roskoski, 2015, 2019b). In addition to these, another motif on the C-lobe is highly conserved suggesting it plays an important role in the catalysis—HRD (rarely YRD) motif. The aspartate residue of this motif is required for the orientation of the hydroxyl group of the substrate peptide at the P-site and the transfer of the phosphoryl group. Arginine residue interacts with the phosphorylated activation segment thereby contributing to its correct orientation. Histidine (or in rare cases tyrosine) is considered to be involved in the maintenance of the conserved rigid organization of the catalytic core (La Sala et al., 2016).

The main differences between tyrosine kinases and serine/threonine kinases are found in the protein-substrate binding site. In serine/threonine kinases, the phosphorylatable serine or threonine of the protein substrate interacts with backbone residues near the end of the activation segment. Basic residues of the protein-substrate N-terminal interact with surface acidic residues of the C-lobe. Additionally, peptide substrate is fixed by serine in the glycine rich loop and lysine in the catalytic loop and also to threonine in the P+1 loop. These three residues are highly conserved in the majority of protein-serine/threonine kinases, and they are positioning the target hydroxyl group of a substrate in the catalytic cleft (near the γ-phosphate of ATP) where the phosphotransfer reaction happens (P-site). Since both serine and threonine hydroxyls are linked to the β-carbon, they have similar mechanisms of the catalysis. On the other hand, in tyrosine kinases, after DFG motif, there is a very stable region that contains the three tyrosine phosphorylation sites. The protein substrate chain positions in a manner that one of the tyrosines is oriented with its hydroxyl group lying in phosphorylation site P-site. The following tyrosine residue lies in the P+1 site. Proline residue interacts with the tyrosyl residue of the protein-substrate and is responsible for positioning the P-site tyrosine in the phosphotransfer site. The tyrosine ring is also positioned by Arg (Hubbard et al., 1994; Taylor et al., 1995; Roskoski, 2015).

**Figure 3:**
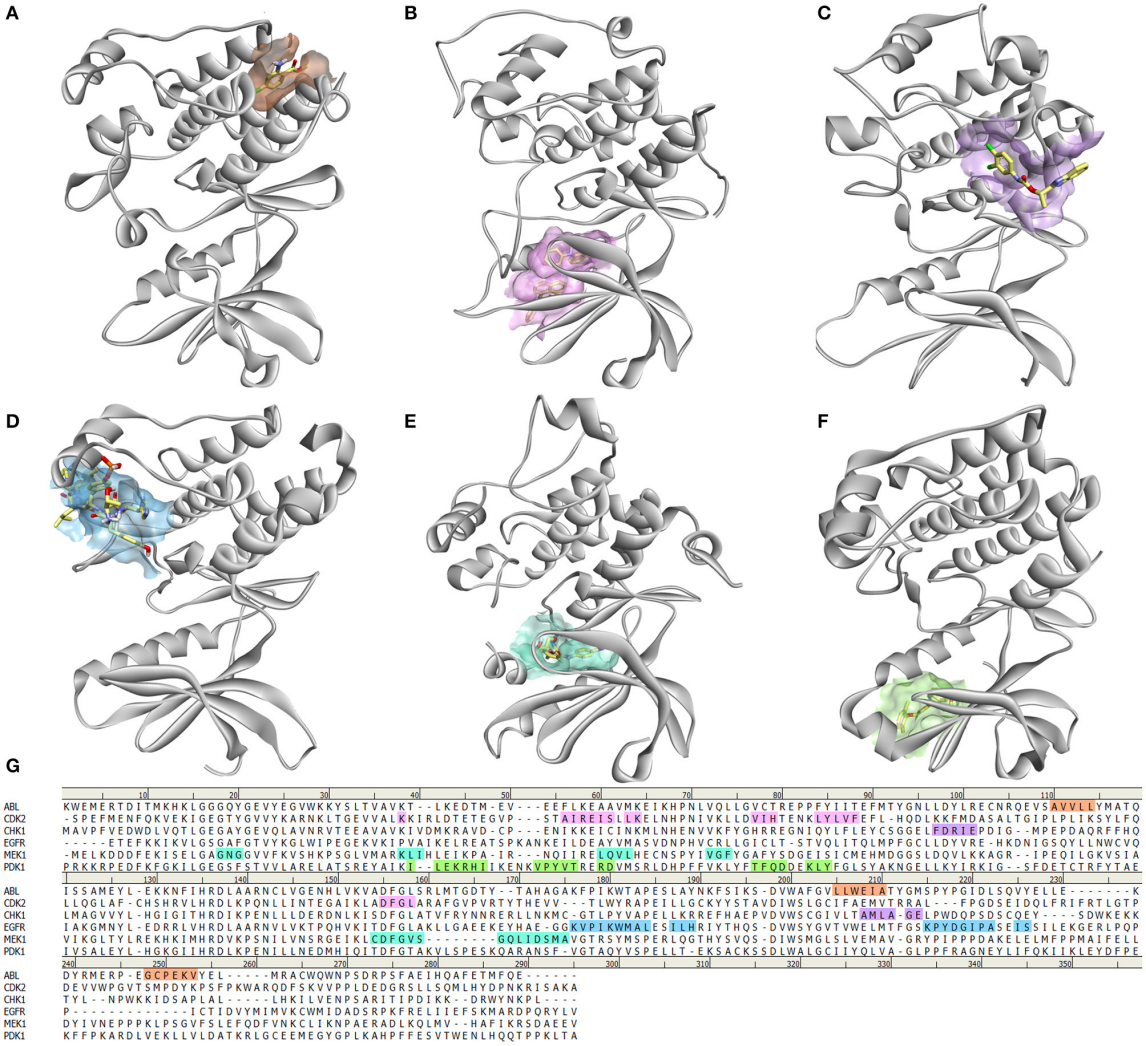
Diversity of allosteric binding pockets in different protein kinases. **(A)** ABL kinase in complex with fragment FRAG1 (PDB: 3MS9) (Jahnke et al., 2010); **(B)** CDK2 in complex with 2 molecules of 8-anilino-1-naphthalene sulfonic acid (PBD: 4EZ7) (Martin et al., 2012); **(C)** CHK1 bound to allosteric inhibitor (1S)-1-(1H-benzimidazol-2-yl)ethyl (3,4-dichlorophenyl)carbamate (PDB: 3JVR) (Vanderpool et al., 2009); **(D)** EGFR in complex with Mig6 protein (PDB: 4R3P) (Park et al., 2015); **(E)** PDK1 with PIF pocket inhibitor RF4 (Rettenmaier et al., 2015); **(F)** MEK1 in complex with 5-bromo-N-(2,3-dihydroxyprpoxy)-3,4-difluoro-2-[(2fluoro-4-iodophenyl)amino]benzamide (PDB: 1S9J) (Ohren et al., 2004); **(G)** Sequence alignment of these kinases showing which amino acids are involved in the binding of allosteric modulators.

Moreover, many protein kinases are regulated by different mechanisms such as dimerization, binding of allosteric effectors, or other modifications important for subcellular localization that can modulate their activity. Binding of an allosteric modulator leads to conformational changes that mostly involve structural reorganization of the activation loop, making it a primary end point of allosteric regulation. Effectors or regulatory subunits bind outside the catalytic site, causing the changes in loop conformation through conformational changes of other substructural elements. In most of the cases, regulators bind the αC helix at different locations, allowing control of catalysis from distal regions (Shi et al., 2006). Nevertheless, the αC helix is not the only allosteric binding site, in fact, they are very diverse (Figure 3; Ohren et al., 2004; Vanderpool et al., 2009; Jahnke et al., 2010; Martin et al., 2012; Park et al., 2015; Rettenmaier et al., 2015; Ung et al., 2018). Therefore, understanding the diversity of allosteric regulatory sites among the kinase superfamily gives a unique opportunity for the creation of novel selective allosteric kinase antagonists (Lamba and Ghosh, 2012).

### Active/Inactive States

**Figure 4:**
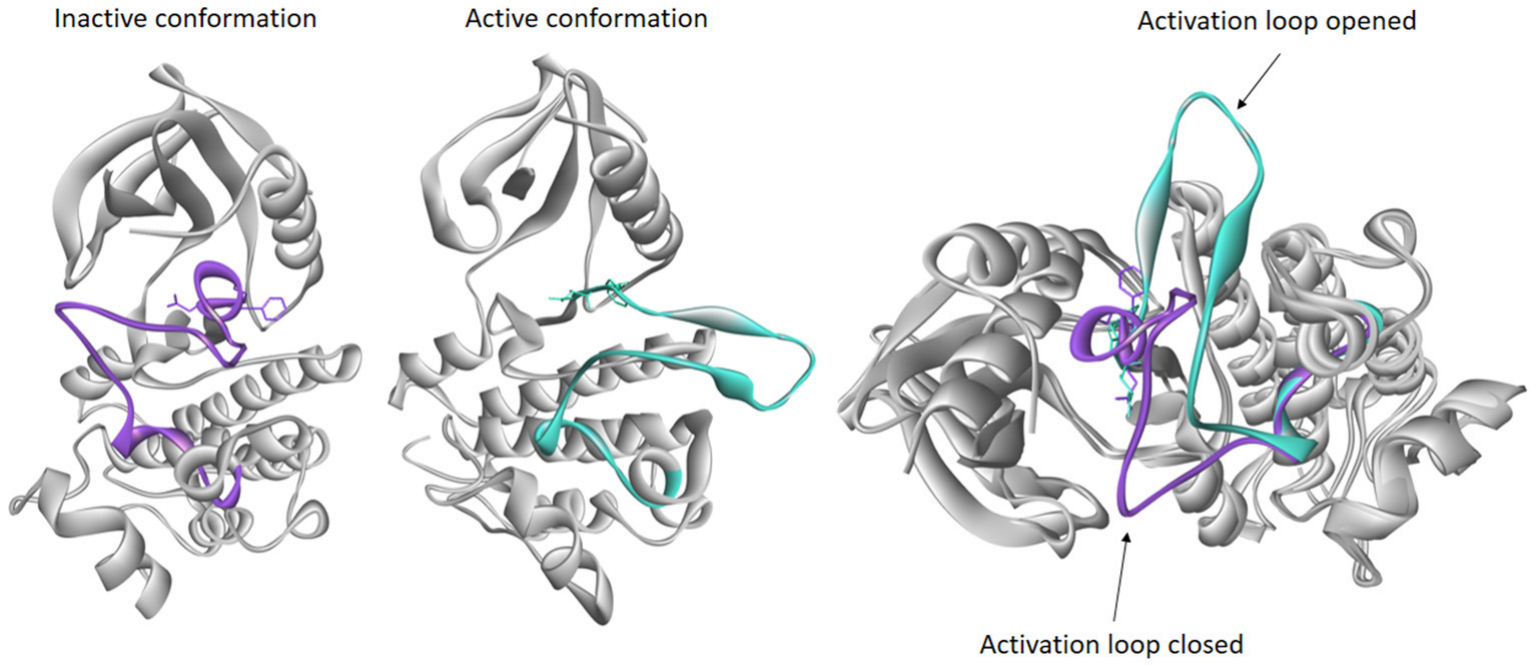
Examples of inactive and active conformations of epidermal growth factor receptor (EGFR) kinase (PDB accession codes: 4HJO and 2GS6, respectively), and their superimposition. The activation loop of inactive conformation (purple) is closed therefore blocking the substrate to enter catalytic loop, while activation loop in active conformation (turquoise) is opened allowing the ATP to bind.

Basically, protein kinases reside in one active state and multiple inactive states (Figure 4). In active kinase conformation, activation loop forms a cleft that binds the substrate. When the substrate peptide binds, it interacts with the HRD motif (His-Arg-Asp). Asp from the DFG motif binds a magnesium ion that interacts directly with an oxygen atom of the β phosphate of ATP. This is followed by formation of a salt bridge between the Glu from the C-helix with a Lys residue in the β3 strand. When the salt bridge is formed, the lysine side chain forms hydrogen bonds with oxygen atoms of α and β phosphates of ATP. The Glycine-rich Loop of the N-lobe stabilizes the phosphates of the bound ATP molecule during catalysis (Taylor and Kornev, 2011; Modi and Dunbrack, 2019). In an inactive conformation, usually the activation loop is blocking the substrate binding, and DFG motif is incompatible with the binding ATP and magnesium ion required for catalysis. Many attempts have been made in order to achieve classification for these conformations and to study interaction of inhibitors in different states (Mobitz, 2015; Ung et al., 2018; Modi and Dunbrack, 2019), and they are all based on the position of highly conserved DFG motif.

The most recent classification was published by Modi and Dunbrack. They have divided kinase structures into three clusters based on the spatial position of the DFG-Phe side chain into DFG-in, DFG-out, and DFG-inter (intermediate) conformations. Each of these three clusters was further divided based on the dihedral angles required to place the Phe side chain, resulting in total of eight clusters: six for DFG-in and one cluster each for the DFG-out and DFG-inter groups. DFG-in represents the DFG motif orientations where DFG-Phe is packed against or under the C-helix. It contains many conformations, among all the typical DFG-in active conformation belongs to this group. DFG-out represents the structures where DFG-Phe is moved into the ATP binding pocket. DFG-inter represents the conformations in which the DFG-Phe side chain is out of the C-helix pocket but has not moved completely to a DFG-out conformation. Usually in this conformation DFG-Phe is pointing upward toward the β-sheets while dividing the active site into two halves (Modi and Dunbrack, 2019). This classification offers insight into active and inactive kinase conformations which are of great importance in structure-based design of kinase inhibitors.

### Types of Kinase Inhibitors

Many reviewers have categorized KIs based on their binding modes into three classes, labeled as types I, II, and III kinase inhibitors (Roskoski, 2016; Bhullar et al., 2018). Type I inhibitors, such as gefitinib, bind to the active DFG-in conformation of a kinase in the phosphorylated ATP catalytic site, and they usually contain a heterocycle that mimic the purine ring of ATP. Considering that the ATP active site is highly conserved among different protein kinases, these ATP-competitive inhibitors display low selectivity profile which may lead to off-target side effects. While the physiological relevance of many off-target effects is still unclear, it was demonstrated that the lack of selectivity is connected with preclinical and clinical cardiotoxicity of kinase inhibitors (Force and Kolaja, 2011; Yang and Papoian, 2012). Possible mechanism behind the KI induced cardiotoxicity lies in binding of these drugs to colony-stimulating factor 1 receptor (CSF1R) (Hasinoff, 2010). Type II inhibitors, such as imatinib, bind to the inactive (DFG-out) conformation of a kinase in the unphosphorylated ATP catalytic site (Dar and Shokat, 2011). Zuccotto introduced type I½ inhibitors as compounds that bind to active ATP catalytic site as type I inhibitors but elongate into the back cavity of the ATP site giving rise to interactions specific for type II pharmacophore. These inhibitors represented by dasatinib, lapatinib, and vemurafenib, display higher selectivity profile as compared to Type I KIs (Zuccotto et al., 2010). Types III and IV are allosteric inhibitors that bind outside the ATP-binding site. Type III includes trametinib that binds to the allosteric site close to the ATP pocket, whereas Type IV inhibitors bind to a pocket distant from the ATP-binding site. Bivalent inhibitor spanning two regions of the protein kinase is termed as Type V (Wong et al., 2017), while KIs that form an irreversible covalent bond with the catalytic site represent Type VI inhibitors (afatinib and ibrutinib) (Roskoski, 2016).

### Developing and Overcoming Resistance to Kinase Inhibitors

Despite the significant advances achieved by the use of protein kinase inhibitors, drug resistance remains one of the greatest challenges toward successful cancer treatment. Various mechanisms can underpin the development of resistance to KIs, including alterations in protein kinases, aberration of downstream pathways, or bypass mechanism that activates parallel signaling pathways (Holohan et al., 2013). Mutations of Bcr-Abl kinase domain were found in over 90% of patients with CML who relapsed after an initial response to imatinib. These mutations include different amino acid substitutions at the active site residues or changes in the kinase flexibility that impair its ability to adopt the inactive conformation required for optimal imatinib binding (Shah et al., 2002). Dasatinib is a novel Abl kinase inhibitor that can bind to both the active (mutated) and inactive (normal) conformations of Bcr-Abl, and its activity has been demonstrated in all imatinib-resistant CML patients, with the exception of those with the T315I mutation that prevents formation of critical hydrogen bond (Shah et al., 2004; Burgess et al., 2005). Docking of dasatinib to three Bcr-Abl conformations (active, inactive, and intermediate inactive) showed that dasatinib binds preferentially to an active conformation, and that binding affinity significantly decreases when the kinase adopts inactive conformation (Laurini et al., 2013). Drug combinations targeting different upstream and downstream components within a single pathway, or targeting parallel kinase pathways, have been proved in clinical trials as an efficient method to overcame or delay therapeutic resistance. For instance, treatment with dabrafenib, a selective BRAF inhibitor, and trametinib, a selective MAPK kinase inhibitor, significantly improved progression-free survival of melanoma patients (Flaherty et al., 2012).

## *In silico* Methods Used In Drug Design

Since the approval of imatinib in 2001, protein kinases have received significant attention from academic and pharmaceutical companies, reflected in a large number of publications, solved crystal structures, and identified small molecule inhibitors for about one-fifth of the human kinome (Wu et al., 2015b). Considerable progress in this field is much owed to the use of computational methods that were able to provide valuable information on structural characteristic of both the kinase and the ligand that are important for favorable interaction and desired inhibitory activity (Agafonov et al., 2015). To design inhibitors for protein kinases it is necessary to understand the structure and dynamics of these enzymes, substrate recognition, and reaction of phosphorylation, product release as well as differences between active and inactive conformations.

There are two main approaches within the framework of computer-aided drug design (CADD): structure-based drug design (SBDD), and ligand-based drug design (LBDD). SBDD is based on structural information gathered from biological targets and includes *in silico* methods such as molecular docking, structure-based virtual screening (SBVS), and molecular dynamics (MD). In contrast, in the absence of information on targets, LBDD relies on the knowledge of ligands that interact with a specific target, and these methods include ligand-based virtual screening (LBVS), similarity searching, quantitative structure-activity relationship (QSAR) modeling, and pharmacophore generation (Ferreira et al., 2015). Over the last years, a large number of studies have reported successful use of CADD in design and discovery of new drugs (Lu et al., 2018b). In this study we provide the comprehensive review of computational tools that led to discovery, design and optimization of KIs as anticancer drugs.

### Ligand-Based Methods in Drug Design

QSAR modeling involves the formation of a mathematical relationship between experimentally determined biological activity and quantitatively defined chemical characteristics that describe the analyzed molecule (descriptors) within a set of structurally similar compounds. The QSAR concept originated in the 1860s, when Crum-Brown and Fraser proposed the idea that the physiological action of a compound in a particular biological system is a function of its chemical constituent, while the modern era of QSAR modeling is associated with the work of Hansch et al. in the early 1960s (Hansch et al., 1962). The aim of the QSAR modeling is to utilize the information on structure and activity obtained from a relatively small series of data to ensure that the best lead compounds enter further studies, minimizing the time and the expense of drug development process (Cherkasov et al., 2014).

Classical 2D-QSAR models correlate physicochemical parameters, such as electronic, hydrophobic or steric characteristics of compounds, to biological activity, while the more advanced 3D-QSAR modeling adds quantum chemical parameters. One of the first approaches used in deriving 3D-QSAR models was CoMFA (comparative molecular field analysis). With this analysis, molecules were described with electrostatic and steric fields, which were correlated to biological activity by means of partial least squares regression (PLS) (Cramer et al., 1988). In addition to the steric and electrostatic descriptors, another approach used in deriving 3D-QSAR models was Comparative Molecular Similarity Index Analysis (CoMSIA). CoMSIA approach additionally uses three novel fields comparing to CoMFA, describing the ligand's hydrophobic properties, the presence of the hydrogen bond donors (HBD), and the presence of hydrogen bond acceptors (HBA) (Klebe et al., 1994). The main limitation of the CoMFA/CoMSIA methods is that they are largely dependent on the alignment of 3D-molecular structures which is often a slow process prone to subjectivity. Recently, modern QSAR programs that use new generation of 3D-descriptors, so-called grid-independent (GRIND) descriptors, have been developed and used for multivariate analyses and 3D-QSAR modeling (Pastor et al., 2000; Duran et al., 2009; Smajić et al., 2015; Gagic et al., 2016b).

Recent cases of reported QSAR studies aimed at providing useful information to guide the discovery of new potent KIs are listed in Table 2. Some of them will be discussed in this chapter.

**Table 2:** Selected studies that have used QSAR in the design of kinase inhibitors.

**Target kinase**	**QSAR method**	**Software package**	**References**
Mer	3D	Pentacle	Shiri et al., 2016
Lyn	2D	JMP	Naboulsi et al., 2018
HER2, EGFR	3D	SYBYL	de Angelo et al., 2018
EGFR	2D and 3D	SYBYL	Simeon et al., 2019
IKK-β	2D and 3D	Discovery studio; Schrödinger suite	Wang et al., 2019a
EGFR	3D	SYBYL	Zhao et al., 2019a
Src	3D	Vlife MDS	Koneru et al., 2019
VEGFR-2	3D	MOE	Mohamed et al., 2019
PKMYT1	2D	MOE	Najjar et al., 2019

Koneru et al. have used QSAR combined with molecular dynamics to redesign second-generation Src kinase inhibitor RL-45 in order to withstand the gatekeeper residue mutation and enhance binding affinity. They integrated fragment-based drug discovery (FBDD) technique with QSAR and molecular dynamics to assess novel Src kinase inhibitors. Newly designed compounds were assumed to be able to mitigate mutation-related Src kinase resistance and to bind more efficiently to the kinase active site and were proposed for further synthesis (Koneru et al., 2019). Wang et al. applied QSAR studies on a series of 2-acylamino-3-aminothienopyridine analogs in order to design new IKK-β inhibitors (Wang et al., 2019a). Obtained information on physicochemical, structural, electrostatic, and steric properties revealed that bulky aryl substituents at position C3 on the piperidine ring have favorable effect on activity, which led to the design of an in-house library. Compounds with best predicted activities were further subjected to docking studies. Based on these results two new compounds B01 and B02 were identified as potential IKK-β inhibitors, with predicted pIC_50_ activities of 7.18 and 7.17, and binding affinities of 41.6 and 40.1 kcal/mol, respectively.

Comparative 2D- and 3D-QSAR studies, followed by molecular docking were conducted on a series of quinazoline derivatives acting as EGFR inhibitors (Noolvi and Patel, 2013). According to the 2D-QSAR multiple linear regression (MLR) model, anticancer activity of quinazoline derivatives was influenced by lipophilicity and number of hydrogen bond donors. Presence of short chain ethers such as methoxy-, ethoxy- at C-6 and C-7 positions of quinazoline was found favorable for the activity, while N-containing groups should not be directly attached to the quinazoline ring. 3D-QSAR kNN-MFA (k-nearest neighbor molecular field analysis) revealed that the presence of electronegative groups on the anilino moiety site, electropositive groups at position C7, and a bulky aromatic substituent at C4 increases the EGFR kinase inhibitory activity.

Virtual screening (VS) refers to a group of *in silico* methods widely used in drug discovery to search large-scale compound databases in order to select a more manageable number of candidates with the highest probability of displaying the desired biological activity (Gagic et al., 2016a; Oluic et al., 2017; Vucicevic et al., 2017; Banegas-Luna et al., 2018). This method has been very popular among pharmaceutical companies since it enables developing drugs in time and cost-effective manner and increases the chance of selected candidates to reach clinical studies. Considering the constant improvement of computational power, it is expected that in the near future VS will be a reasonable alternative to high throughput screening (HTS) (Kumar et al., 2015). There are generally two approaches to screen molecular libraries: LBVS that will be discussed in this section and SBVS.

LBVS is often applied when there are known active compounds, but the target of action is not known, or the crystallographic structure of the protein is not available. These active compounds are then used as ligands to screen molecular libraries based on the similar property principle, which states that structurally similar compounds should possess similar biochemical properties (Nikolic et al., 2015; Bajorath, 2017). For each compound from the virtual library, the similarity with the known active is calculated. Many different strategies for measuring similarity have been developed, including Cosine coefficient, Euclidean distance, Soergel distance, and Tanimoto coefficient (Bajusz et al., 2015). Compounds are ranked based on the similarity score and those at the top are selected as virtual hit molecules for further optimization and synthesis. Modern VS protocols include additional filtering steps in order to exclude compounds that e.g., have low similarity score, do not fall within the Lipinski's rule of five, are not feasible for synthesis or are not available for purchase (Neves et al., 2018).

Besides similarity searches, pharmacophore search is one of the most commonly used LBVS techniques. Given a list of known actives, pharmacophore model can be derived to define the minimum structural requirements that molecule must possess in order to exhibit good activity profile (Vittorio et al., 2019). It is then possible to search large databases, such as PubChem (Kim et al., 2019), ChEMBL (Mendez et al., 2019), and DrugBank (Wishart et al., 2018), for identification of lead compounds that fit to the pharmacophore structure (Bacilieri and Moro, 2006). Several studies that describe the use of LBVS methodology in discovery of potential kinase inhibitors have been listed in Table 3. Pharmacophore-based VS model was employed to search for new tumor progression locus-2 (Tpl2) inhibitors (Teli and Rajanikant, 2012). Tpl2 is a serine/threonine kinase in the MAPK signaling pathway that regulates cell proliferation, survival, and death and participates in many processes of tumor development (Lee et al., 2015). For this purpose, Asinex database was screened using PHASE 3.0 module of the Schrodinger molecular modeling software which resulted in six potential Tpl2 kinase inhibitors. A 3D QSAR pharmacophore model was developed from the structures of known inhibitors of MAPK1 (ERK2) and used for virtual screening of ZINC database (Irwin et al., 2012) that contains over 750 million compounds, DrugBank with 13,443 drugs (Wishart et al., 2018), NCI (https://cactus.nci.nih.gov/ncidb2.2/) with 250,250 structures, Maybridge (https://www.maybridge.com) with over 53,000 compounds and Chembank database (Seiler et al., 2008). Top screened compounds were then subjected to molecular docking that identified new scaffolds with high potency and selectivity against ERK2 (Larif et al., 2014).

**Table 3:** Selected studies that have used LBVS in the design of kinase inhibitors.

**Target kinase**	**Software package**	**References**
EGFR	PHASE	Sudha et al., 2015
CDK2	SYBYL	Zhang and Ren, 2018
ERK-1/2	QSAR-Co	Halder et al., 2019
VEGFR 2	Discovery studio	Sobhy et al., 2019
ALK	PHASE	James et al., 2019
CDK9/Cyclin T1	LigandScout	Hussain and Verma, 2019
FGFR1	Discovery studio	Liu et al., 2020

It can be concluded that VS strategies, especially Pharmacophore-based VS and combined use of VS and molecular docking, can be a reliable tool for future discovery of new KIs and have a potential to replace a HTS that is costly and time consuming process.

#### Case Studies

##### Application of quantitative structure-activity relationship in structure elucidation of Lyn kinase inhibitors

The generalized linear model (GLM) and the artificial neural network (ANN) QSAR models were combined with structural analysis in order to define pharmacophore of Lyn kinase inhibitors (Naboulsi et al., 2018). Lyn kinase is a member of the Src family of tyrosine kinases that was found to be correlated with chemotherapeutic resistance of cancer cells in patients with CML (Chakraborty et al., 2013; Aira et al., 2018). Derived pharmacophore for the inhibition of Lyn kinase suggested the presence of planar heterocyclic ring that contains HBD and HBA, a spacer that allows free bond rotation and central hydrophobic area that is linked to the aromatic ring substituted with lipophilic groups. These structural futures can be found in nilotinib and dasatinib that are approved for treatment of CML (Figure 1). Pyrimidine moiety of nilotinib has the role of the hydrogen bonding region; the attached amino group serves as a spacer that is linked to hydrophobic benzyl group connected with another aromatic ring that is substituted with lipophilic trifluoromethyl group and methylimidazole. Aminopyrimidine moiety is also present in dasatinib that is indicated in CML patients that developed resistance to nilotinib (Okabe et al., 2011). Dasatinib, instead of central hydrophobic benzene ring, contains thiazole connected to an aromatic ring with lipophilic substituents. Results of these QSAR studies can be of great help in future design and lead to optimization of new, more potent Lyn kinase inhibitors for treatment of patients with imatinib and nilotinib-resistant CML.

##### Quantum mechanical based quantitative structure-activity relationship of N-phenylquinazolin-4-amine derivatives as epidermal growth factor receptor inhibitors

Recently, Simeon at al. applied several 2D- and 3D-QSAR methodologies on a series of EGFR inhibitors, derivatives of N-phenylquinazolin-4-amine (Simeon et al., 2019). 2D QSAR models were created using physico-chemical descriptors, e-state indices and molecular fingerprints, while 3D-QSAR models were developed using CoMFA, CoMSIA, and quantum mechanical (QM) methods. Based on the calculated statistical parameters, the QM-QSAR model displayed better predictive power compared to the other models. Development of this model started with docking of N-phenylquinazolin-4-amine analogs to the EGFR active site and calculation of pairwise interaction energies between each inhibitor and amino acid residues using quantum mechanics. Distances that hold information about the position of the quinazoline ring and the aniline pharmacophores within the active site of the EGFR were extracted and used as descriptors for the QM-QSAR model. Combined 2D- physico-chemical and QM-QSAR model showed even better predictivity and provided more precise information about structural characteristics that are important for EGFR inhibitory activity. Based on the results of this study, it can be concluded that a combination of classical and more advanced quantum mechanical QSAR methodologies represents a good concept for future design of new EGFR inhibitors.

##### Discovery of potential FGFR1 inhibitors using pharmacophore-based virtual screening

Pharmacophore-based VS protocol was developed in Maestro 9.0 software package (https://www.schrodinger.com/) and used to screen SPECS database (http://www.specs.net) for potential FGFR1 inhibitors (Zhou et al., 2015). Database was previously filtered to extract only compounds with drug-like properties that comply with the Lipinski's rule of 5. Activities of top ranked compounds were predicted with constructed atom-based 3D-QSAR model, and those with highest activities were purchased for experimental enzyme assay. Nineteen hits exhibited moderate inhibitory activity with more than 50% FGFR1 inhibition at 50 μM concentration and IC_50_ values of most active compounds were 7.9 and 55.5 μM. It should be mentioned that the identified compounds had low structural similarity with previously reported FGFR1 inhibitors and offered novel chemical scaffolds for future optimization of FGFR1 inhibitors.

##### Structure based methods in drug design

Recent progresses in the field of X-ray crystallography, Nuclear Magnetic Resonance (NMR) techniques, and cryo-electron microscopy (CEM) caused a significant increase in the number of known 3D structures of proteins (Sun et al., 2011). With known 3D structures of proteins, docking became a method of choice in drug design.

Molecular docking predicts the most probable orientation of one molecule toward another (Lengauer and Rarey, 1996). It can be performed between a small molecule and a target protein (ligand-protein docking) or between two proteins (protein-protein docking). In ligand-protein docking, which will be discussed here, the samples of conformations of small molecules–ligands are placed into the binding sites of protein, where scoring functions are used to calculate which of these conformations best fits the target protein binding site (Sousa et al., 2006; Warren et al., 2006). Overall, docking protocols include search algorithm and a scoring function. Initially, the search algorithm is used to orient small molecules in the target binding site (Taylor et al., 2002). Sampling of conformational space has to be carried out with acceptable accuracy to determine the conformation that best fits the binding site, but fast enough to evaluate a large number of docked ligands. With today's computer power it would be impossible to explore all the degrees of freedom for ligand and protein complex. Therefore, there are different ways to overcome this problem. Search algorithms can be systematic and stochastic and deterministic (Novič et al., 2016). Systematic search algorithms sample the search space at predefined intervals while stochastic make random changes until a user-defined termination criterion is met, and because of that outcome can vary (Morris and Lim-Wilby, 2008). Search algorithm is then followed by scoring function that estimates the affinity of ligand through the assessment of interactions between ligands and potential targets (Kitchen et al., 2004). Scoring functions can be physics-based, empirical, knowledge-based, and machine learning-based (Liu and Wang, 2015; Li et al., 2019). The physic-based scoring function computes the free energy of binding by summing up the van der Waals and electrostatic interactions between the protein–ligand (enthalpy), and adding the torsion entropy of ligand as well as the solvation/desolvation effect described by explicit and implicit solvent models (Huang et al., 2006; Liu and Wang, 2015). Empirical scoring function estimates the binding affinity of a complex by accumulating significant energetic factors for protein–ligand binding (hydrogen bonds, hydrophobic effects, steric clashes, etc.). It uses a training set with known binding affinities of protein–ligand complex and optimizes the weights of the energetic factors by the means of regression analysis (Eldridge et al., 1997; Liu and Wang, 2015). The knowledge-based scoring functions also uses structural information of large set of known protein–ligand complexes and converts it into distance-dependent Helmholtz free interaction energies (Muegge and Martin, 1999; Li et al., 2019). Machine-learning based scoring functions for docking are getting more interests nowadays. These methods combine QSAR analysis and protein–ligand interaction evaluation. They combine QSAR analysis and protein–ligand interaction evaluation. The training set of protein–ligand complexes with known structures and binding affinities is required for a model calculation. Structural interaction fingerprints between a protein and a ligand are coded with certain descriptors (electrostatic interactions, hydrogen bonds, or aromatic stacking, surface or shape properties, molecular weight, number of rotatable single bonds, etc.). Then, different machine-learning algorithms are employed for variable selection (Deng et al., 2004; Zhang et al., 2006).

Molecular docking can be employed in many parts of drug discovery process, such as structure–activity studies, lead optimization, structure based virtual screening, binding modes defining, chemical mechanism studies, etc. (Nikolic et al., 2013; Bautista-Aguilera et al., 2014; Oluic et al., 2017; Albert et al., 2019). Most popular docking programs are DOCK (Kuntz et al., 1982), Autodock (Morris et al., 2009), AD Vina (Trott and Olson, 2010), GOLD (Verdonk et al., 2003), GLIDE from Schrödinger suite (Halgren et al., 2004), and they mostly differ in search algorithms and scoring functions they use. It is always recommendable to explore several different docking programs and then decide on the best one for the specific protein-ligand complexes.

For the last decade, molecular docking has been widely used in design of protein kinase inhibitors (Table 4). Tsou et al. designed 4-(phenylaminomethylene) isoquinoline-1, 3(2H, 4H)-dione derivatives, an original class of potent inhibitors that selectively inhibit CDK4 over CDK2 and CDK1 activities. They used SAR and docking to identify interactions between the ligands and residues of the protein's ATP binding pocket and to find interactions with amino acids unique to CDK4 (His82, Val83, and Asp84) and to optimize compounds with improved activity and selectivity toward CDK4 (Tsou et al., 2008). Gopalsamy et al. identified a compound as B-Raf inhibitor from high throughput screening (HTS) and used docking into the crystal structure of B-Raf-Sorafenib complex (1UWH) (Wan et al., 2004) to identify important protein–ligand interactions (two hydrogen bonds with Glu500 and Asp593, and hydrophobic interactions with Ile462, Trp530, Phe582, Ile 512, His 573, and Ile 571) and to optimize the scaffold to obtain compound with improved potency (Gopalsamy et al., 2009). In 2018, Amr et al. synthetized a series of macrocyclic pyrido-pentapeptide candidates, and identified their activity *in vitro* on several kinases. Following docking study of the best compound into VEGFR-2, EGFR, PDGFR, provided information of the binding mode and important protein-ligand interactions which can be further used as a guideline for future design (Amr et al., 2018). In their efforts to design 2-phenazinamine derivatives as Bcr-Abl tyrosine kinase inhibitors, Kale and Sonwane combined molecular docking studies with G-QSAR (Group-Based QSAR). Their *in silico* studies predicted better activity for the thiazolidones and benzenesulfonyl derivatives of phenazinamines than doxorubicin. However, *in vitro* cytotoxic activity was good, though still less than of doxorubicin (Kale and Sonwane, 2018).

**Table 4:** Selected studies that have used docking in the design of kinase inhibitors.

**Target kinase**	**Software package**	**References**
EGFR	Maestro	Hu et al., 2017
VEGFR-2, CDK-2 and PDGFRβ	MOE	Amr et al., 2018
Bcr-Abl	Autodock	Kale and Sonwane, 2018
PKMYT1	GOLD	Platzer et al., 2018
EGFR, PDGFR-β	GOLD	Fischer et al., 2018
Pim-1	MOE	Mohareb et al., 2019
PAK4	Glide	Gao et al., 2019
Bcr-Abl	Discovery studio	Melge et al., 2019
EGFR	Glide	Debnath et al., 2019
PI3K	AutoDock	Wang et al., 2019b
Pim-1	AutoDock	Hazhazi et al., 2019
VEGFR-2	GOLD	Zhao et al., 2019b
PKC	Glide	Wang et al., 2019c
EGFR	MOE	Khodair et al., 2019

Molecular dynamics (MD) is a simulation technique for studying time dependent evolution of molecular system. Relying on principles of classical mechanics, in MD simulations, positions, and velocities of atoms are computed by classical (Newtonian) laws of motion (Klepeis et al., 2009). The forces acting on these atoms are computed using potential energy functions known as force fields. All common force fields express potential energy through bonded terms (covalent bond-stretching, angle-bending, torsion potential, improper torsions) and non-bonded terms (Lenard Jones repulsion and dispersion and Coulomb electrostatics) (Vanommeslaeghe et al., 2014). Several force fields were found to provide quite accurate representations of the structure and dynamics of a number of small globular proteins on the sub-microsecond timescale (Beauchamp et al., 2012). Most commonly used force fields today are CHARMM (Yin and MacKerell, 1998), AMBER (Weiner et al., 1984; Cornell et al., 1995), GROMOS (Oostenbrink et al., 2004), OPLS (Jorgensen et al., 1996), and COMPASS (Sun, 1998) force fields since they include various chemical groups present in macromolecules and drug-like entities.

Recent algorithmic advances and increase in computational power have enabled simulation studies of protein systems on biophysically-relevant timescales. Combined with modern improvements in the quality of force field parameters, protein structure prediction and modeling has advanced impressively (Beauchamp et al., 2012; Raval et al., 2012; Piana et al., 2014). Providing structural and dynamical insight into the studied molecular system difficult to obtain experimentally, as well as thermodynamics and kinetic understanding of the system, MD simulations are usually referred to as “computational microscopes” (Dror et al., 2012). In this review, we discuss the usefulness of MD and MD-based methods in the discovery of kinase inhibitors through different case studies presented below.

Structure-based virtual screening (SBVS) is based on the knowledge of the 3D structure of the target protein, obtained by X-ray crystallography, NMR, cryo-EM or homology modeling (Lionta et al., 2014). Nowadays, the SBVS methods are enabled thanks to a large number of 3D structural information deposited in the PDB. As described above, by using the 3D structural information of the protein target, we are now able to investigate the basic molecular interactions involved in ligand-protein binding and understand experimental results up to atomic levels. In SBVS, large libraries of commercially available drug-like compounds that are computationally screened against proteins of known structure and those that are predicted to bind well can be experimentally tested (Benod et al., 2013; Vucicevic et al., 2016; Oluic et al., 2017).

#### Case Studies

##### Structure-based design of imidazo [4,5-b]pyridin-2-one-based p38 mitogen-activated protein kinase inhibitors

**Figure 5:**
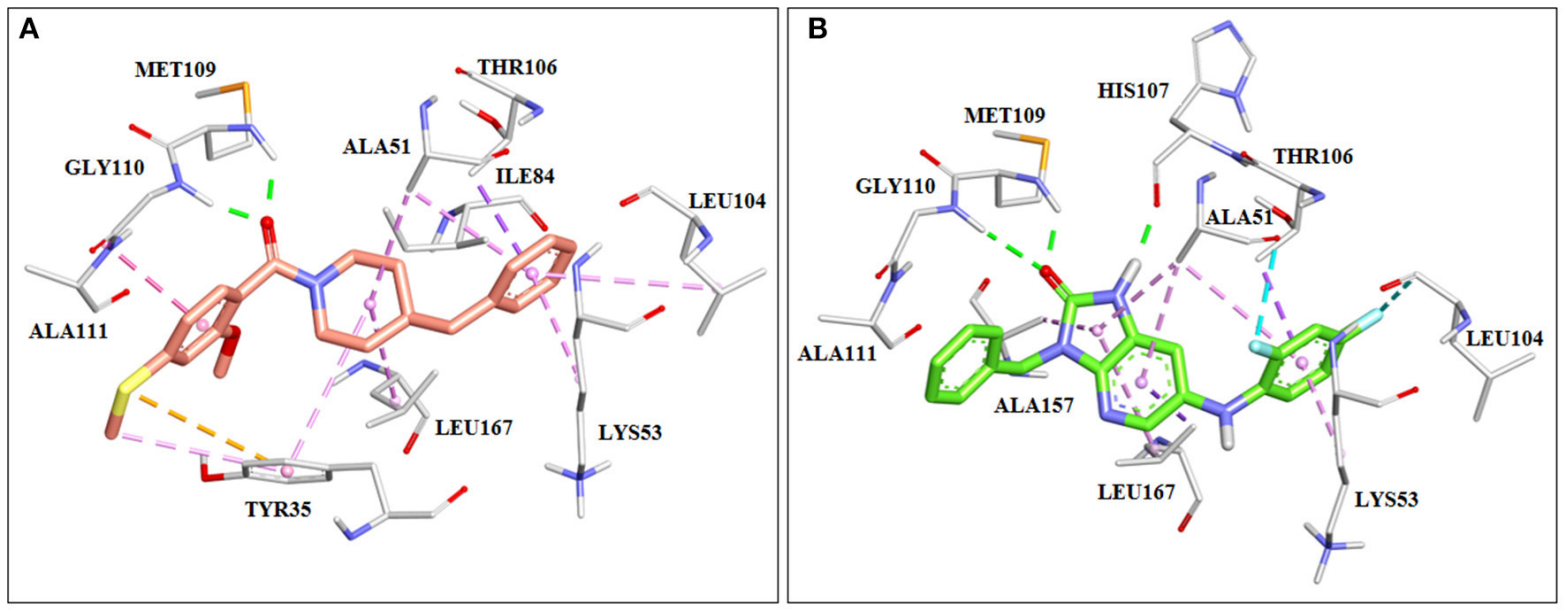
Crystal structures of p38 mitrogen-activated protein (MAP) kinase with imidazo[4,5-b]pyridin-2-one derivatives. **(A)** Lead compound found by HTS (PDB: 6M95). **(B)** Potent p38 MAP kinase inhibitor designed using structure-based drug design (SBDD) approach (PDB: 6M9L).

Using structure-based drug design, Kaieda et al. have identified a series of potent p38 mitogen-activated protein kinase inhibitors. First they identified the lead compound with moderate inhibitory activity toward p38 MAP kinase by means of high-throughput screening. The lead compound was then crystalized with the MAP kinase. The X-ray crystallographic results showed that carbonyl group of the compound forms two hydrogen bonds with the backbone amide of Met109 and Gly110 of the enzyme (Figure 5A). The hinge backbone conformation of their crystal structure was different from that typically seen in protein kinases. Namely, usually the backbone amide and carbonyl group of Met109 are directed toward the ATP binding site and accessible for creation of hydrogen bonding with ligand. In the obtained crystal structure a flip of the peptide bond between Met109 and Gly110 was noticed which led to a switching of the hydrogen-bond acceptor and donor distribution around the peptide plane, instead. It was assumed that this flip could be responsible for the high kinase selectivity. After switching the scaffold of the carbonylpiperidine group while maintaining this binding mode, a series of synthetized imidazo[4,5-b]pyridin-2-one derivatives were identified as potent inhibitors of the p38 MAP kinase (Figure 5B; Kaieda et al., 2019).

##### Discovery of novel Pim-1 kinase inhibitors by support vector machine, pharmacophore modeling and molecular docking

In 2011 Ren et al. reported the discovery of novel potent Pim-1 inhibitors by combining ligand- and structure-based filtering methods. In order to find new molecules, a pipeline was created that consisted of support vector machine-based VS (SVM-based VS), pharmacophore-based VS (PB-VS), and docking-based VS (DB-VS) and screened approximately 20 million molecules. Protocol was evaluated by using the library which contained 203 known Pim-1 inhibitors and around 117,000 generated decoys. For validation of the performance of VS, the percentage of predicted compounds in known inhibitors, percentage of known inhibitors in predicted compounds, as well as enrichment factor were calculated. The combined protocol showed much better performance than solely SB-VS, PB-VS, and DB-VS. Finally, 47 compounds were selected for further *in vitro* Pim-1 kinase inhibitory assay for an inhibitor concentration of 10 μM, and 15 compounds showed nanomolar level or low micromolar inhibition potency against Pim-1. In conclusion, new scaffolds with the potential for the future chemical development were found (Ren et al., 2011).

##### Discovery of pazopanib, vascular endothelial growth factor family of receptor inhibitor

**Figure 6:**
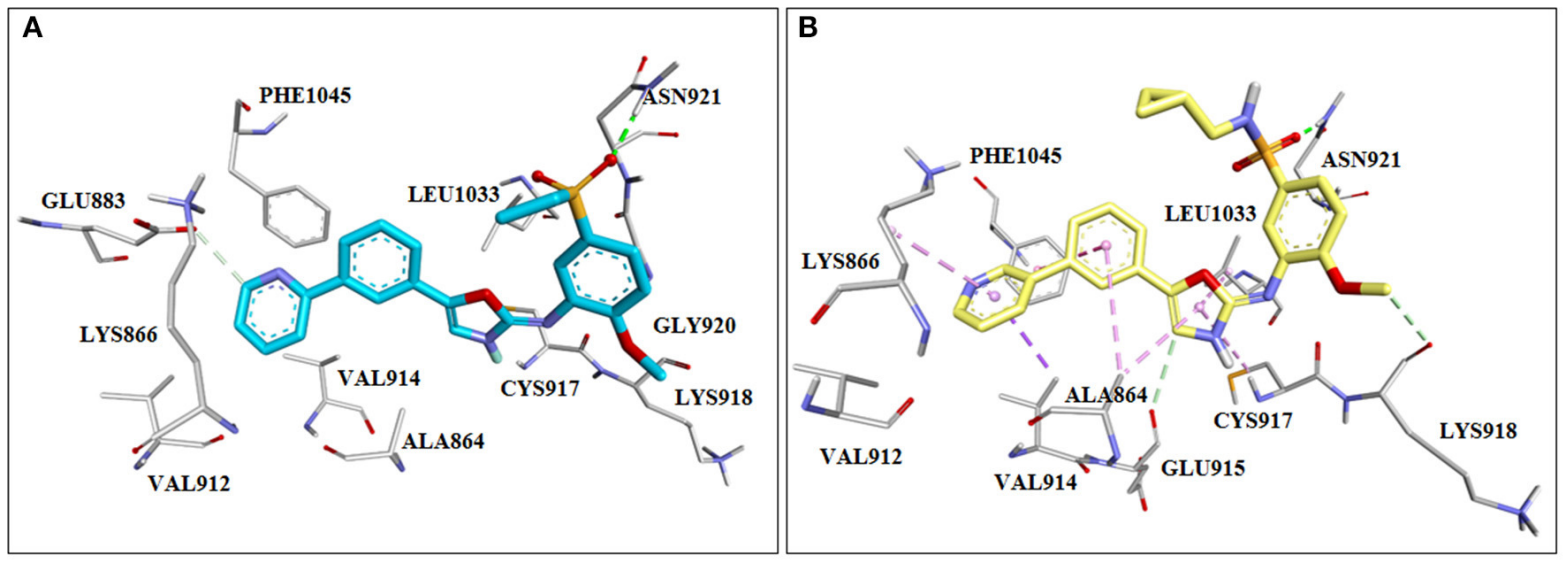
Crystal structures of initial screening hits for inhibitors of the vascular endothelial growth factor (VEGF) that lead to the discovery of pazopanib. **(A)** PDB: 1Y6A, **(B)** PDB: 1Y6B.

In 2008 Harris et al. published a paper explaining their discovery of pazopanib. That was a good example of usage of homology modeling and SBDD in the discovery of a drug that is today on the market. Since the crystal structure of VEGFR2 was not available at that time, a homology model of the VEGFR2 enzyme based on FGFR crystal structures was created to predict the binding mode of dimethoxyquinazoline analogs. It was noticed that the pyrimidine and the quinazoline bound similarly in the ATP binding site, making the hydrogen bonds with the Cys919 of the backbone (Figure 6). Crystallization of these compounds with VEGFR2 confirmed *in silico* results (PDB: 1Y6A, 1Y6B). Finally a series of new analogs was designed, synthetized, and tested *in vitro*, which led to the discovery of pazopanib (Harris et al., 2005, 2008).

##### Rational discovery of dual-indication multitarget phosphodiesterase/ kinase inhibitor

One of the latest studies published this year by Lim et al. combined molecular docking with other bioinformatics tools, with the goal of finding multi-target-multi-indication drugs (Lim et al., 2019). They have used structural and chemical genomics data and combined tools from bioinformatics, chemoinformatics, protein-ligand docking, and machine learning to create a novel structural systems pharmacology platform−3D-REMAP. It used four networks as input: 1. protein–ligand association, 2. off-target, 3. ligand–ligand similarity, and 4. protein–protein similarity. The protein–ligand associations were obtained from ChEMBL, DrugBank, and from other publications about kinome assays (Christmann-Franck et al., 2016; Drewry et al., 2017; Klaeger et al., 2017; Merget et al., 2017) and protein structure-based off-target prediction from binding pocket similarity search and protein–ligand docking. Ligand–ligand similarity was calculated in MadFast software from ChemAxon, and protein–protein similarity was run through BLAST. Moreover, to validate and show advantages of their platform, they searched for marketed drugs that could be dual-indication agents. In their study, they focused on drugs that could reduce the cardiotoxicity of anti-cancer therapy. They predicted that levosimendan, a phosphodiesterase (PDE) inhibitor which is used for heart failure, also inhibits serine/threonine-protein kinase RIO kinase 1 (RIOK1) and several other kinases [Ca2+/calmodulin-dependent protein kinase II (CAMK2), FMS-like tyrosine kinase 3 (FLT3), RIOK3, etc.]. To validate their results they tested anti-cancer activity of levosimendan for more than 200 cancer cell lines. Their experimental results showed that levosimendan is active against several cancers, particularly lymphoma, through the inhibition of RIOK1 and its RNA processing pathway (Lim et al., 2019). Since this study is brand new, the time will tell whether levosimendan will be a candidate for clinical research.

##### Fragment-based drug design of kinase inhibitors

Discovery of kinase inhibitors is a highly competitive process wherein teams of experienced researchers, both from academia and industry, use all the previous knowledge and new ideas to provide more effective therapies for patients. Depending on the available methodologies, one research group may start their drug discovery project with a high-throughput screening (HTS) campaign and search for the bioactive (HIT) compounds against the studied kinase. Selected HIT molecules usually possess drug-like properties and should be further optimized with the aid of lead optimization techniques. Contrary to drug-like molecules, fragments have a smaller number of heavy atoms (HA) and they should comply with Rule of Three (RO3), in which molecular weight is <300 Da, number of hydrogen bond donors and acceptors should be ≤ 3 and clogP is ≤ 3 (Congreve et al., 2003).

Fragment molecules tend to show high micromolar to millimolar affinities for a certain biological target. The advantages of using fragments in drug design studies of novel kinase inhibitors are numerous:

- Fragments displaying affinity to the examined biological target can overcome the entropy barrier and their binding is related to the favorable enthalpy contribution (Murray and Verdonk, 2002);
- Comparing to drug-sized molecules, pharmacokinetic and physicochemical properties of fragments could be more efficiently optimized (Leach and Hann, 2011);
- Drug-sized molecules may suffer from a potential loss of complementarity with the studied targets, whereas the fragments seldom possess functional groups that establish ligand–protein intermolecular clashes (Hann et al., 2001);
- Given all the above, FBDD projects can lead to increased HIT rates and discovery of novel fragments interesting from different points of view (binding affinity, synthetic accessibility, intellectual property).

Historically, first FBDD projects were applied by a technique named “SAR by NMR” (structure-activity relationship by nuclear magnetic resonance) (Shuker et al., 1996). In this paper, authors successfully developed a potent compound with nanomolar affinity to FK506 binding protein (FKBP) by merging two building blocks. Except for NMR, protein-fragment interacting patterns are characterized by other biophysical methods such as X-ray crystallography, surface plasmon resonance (SRC), high concentration screening (HCS) assays, isothermal titration calorimetry (ITC), fluorescence correlation spectroscopy and many more (Sun et al., 2011). The choice of a particular method depends on the previous experience in FBDD projects and also the size of fragment libraries.

Until now, fragment-based drug discovery (FBDD) method resulted in FDA approval of three kinase inhibitors—vemurafenib (Bollag et al., 2012), venetoclax (Deeks, 2016), and erdafitinib (Markham, 2019). These excellent textbook examples of FBDD are developed by different biophysical methods; nevertheless, the present review focuses on various *in silico* techniques frequently used in fragment identification and optimization.

In recent years, experimental screening procedures may be replaced by computational methods to reduce the costs and time for early stages of FBDD project (Alves Avelar et al., 2019; Ruzic et al., 2019). It appears that *in silico* studies may support kinase drug discovery at almost every stage of fragment-based drug design projects. Various ligand-based virtual screening (Giordanetto et al., 2011), structure-based (Warner et al., 2006; Zhao et al., 2012), and quantum mechanical (Machrouhi et al., 2010) techniques have been proved as successful in novel fragment identification. Before running any virtual screening protocol, computational chemists must pay attention to the valid preparation of fragment library database. The fragment library databases should obey the aforementioned Rule of 3 (RO3); additionally, their chemical properties are filtered through certain software which removes possible toxicophores and pan-assay interference compounds (PAINS) (Baell and Walters, 2014). Nowadays, computational chemists may use kinase fragment libraries which may assist faster identification of novel hinge binding motifs. Moreover, fragments that target distal pockets from the ATP binding pocket could be scanned by allosteric kinase library, such as Enamine Allosteric Kinase Library (https://enamine.net).

#### Case Studies

##### Identification of PI3K p110β selective fragment

**Figure 7:**
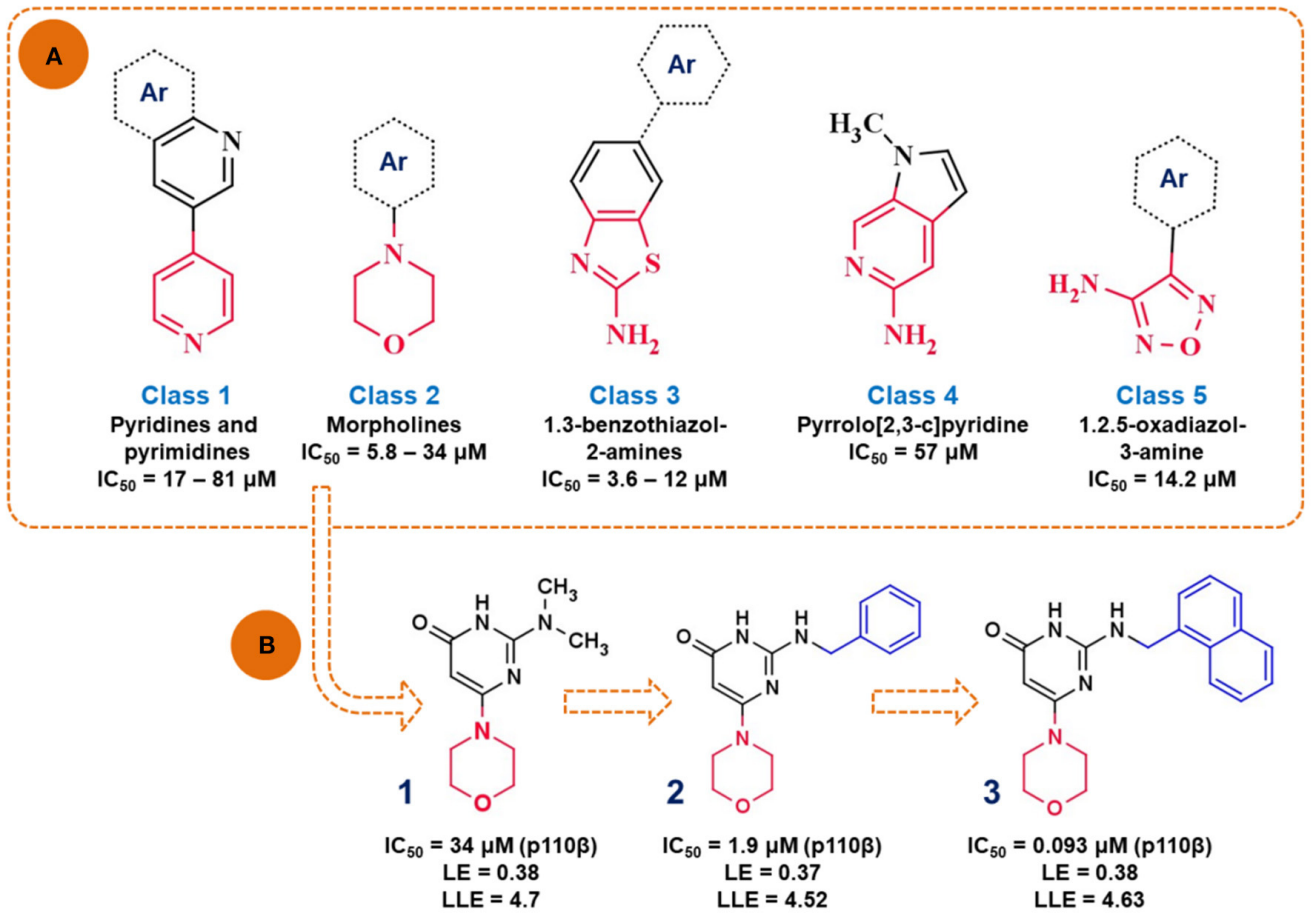
**(A)** Presentation of the identified scaffolds by *in vitro* biochemical screening as PI3K inhibitors (hinge interacting moieties are labeled in red), *Ar*—presents carbocyclic or heterocyclic aromatic rings; **(B)** the scheme of lead optimization of the selected morpholine derivatives.

Intracellular lipid kinases that transfer a phosphate group from ATP to certain cell membrane's phospholipids (Phosphoinositide-4,5-biphosphate, PIP_2_) belong to the family of phosphoinositide 3-kinases (PI3K). These enzymes regulate important cellular events and present interesting drug targets in anticancer drug discovery. Giordanetto et al. (2011) successfully identified fragments that showed selective p110β inhibition. At the time this study was performed, the crystal structure of p110β isoform was not available. Consequently, the homology model was built in MODELLER (Webb and Sali, 2016) by using the crystal structure of p110γ isoform. In this study, authors used AstraZeneca's virtual fragment database and subjected 183,330 fragments to a molecular docking study in GLIDE software (Schrödinger, New York). The poses and orientation of the fragments in the ATP binding pocket were inspected, as well as hydrogen bonding interactions with amino acid residues in the hinge region, affinity and selectivity pocket. The authors reported five chemical classes of fragments (Figure 7A) based on the different heterocyclic rings interacting with the hinge region in p110β and their *in vitro* enzymatic profiles against four human PI3K isoforms (p110α, p110β, p110γ, and p110δ). Overall, the hit rate achieved from this screening was 8.57%, indicating good performance of the molecular docking-based search for novel and chemically interesting fragments as PI3K hinge binders. The authors continued this study with the morpholine derivative, compound (**1**) (Figure 7B), which showed moderate potency against p110β (IC_50_ = 34 μM), but its inhibition of the other p110 isoforms was not determined at the tested concentrations.

In the following study, authors aimed to improve the affinity of the compound (**1**) by substituting the dimethylamino group with a more voluminous 2-(benzylamino) moiety (Giordanetto et al., 2012). The novel compound (**2**) showed improved potency (IC_50_ = 1.9 μM) and efficiency (LE = 0.37 and LLE = 4.52) toward p110β. The rationale for this chemical modification relies on the observation that the bulkier substituents might target amino acid residues M804 and W812 in the proximal selectivity pocket. Finally, compound (**3**) was synthesized by introducing the naphthyl group, which in turn attributed to the nanomolar potency (IC_50_ = 0.093 μM) and improved p110β selectivity profile of compound (**3**).

##### Identification of mitogen-activated protein kinase-interacting kinase 1 inhibitors

Mitogen-activated protein (MAP) kinase interacting kinases 1 and 2 (**MNK1** and **MNK2**) carry out phosphorylation reaction of eukaryotic translation initiation factor 4E (eIF4E) on serine 209 (Wendel et al., 2007). This translation factor is involved in different cellular pathways, such as Ras/Raf/MEK/ERK and PI3-kinase/protein kinase B (Akt) signaling pathways (Proud, 2015). The overexpression of phosphorylated eIF4E leads to several malignant diseases, such as lymphomas, breast cancer, and glioblastoma (Astanehe et al., 2012). The significance of MNK1/2 enzymes in malignant transformation of the cell has led to high demand for drug design of MNK1/2 inhibitors.

One remarkable study was performed in 2010, where Oyarzabal et al. identified a highly potent and efficient fragment entirely by *in silico* modeling. In this comprehensive study, authors combined different virtual screening techniques to identify pharmacological tools for MNK1 inhibition. Initially, the Centro Nacional de Investigaciones Oncológicas (CNIO) database was filtered according to the molecular weight (<300 Da) and calculated solubility values (threshold −4 mol/L). By performing this prefiltering procedure, the authors extracted 42,168 fragment-like compounds for virtual screenings (Oyarzabal et al., 2010).

**Figure 8:**
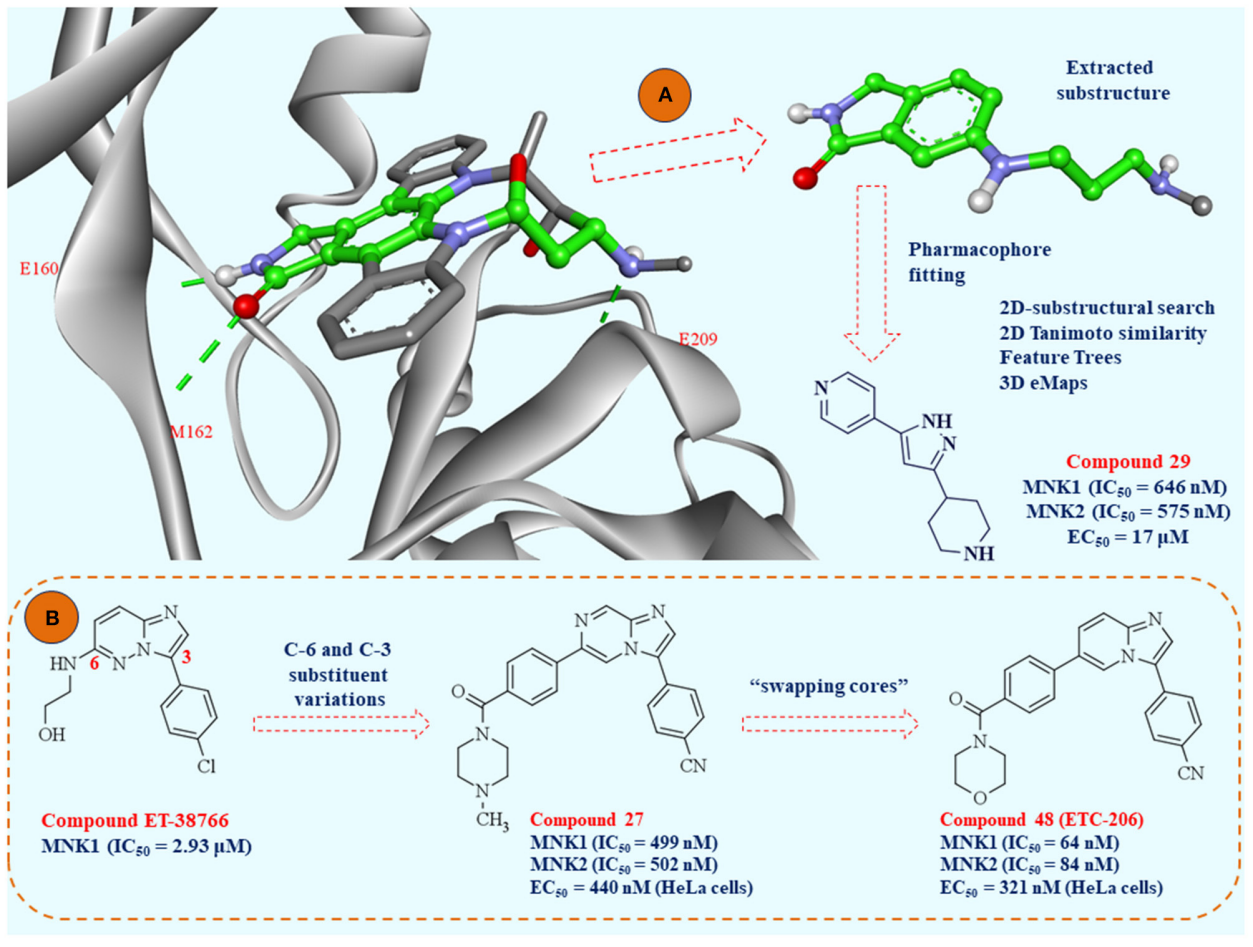
**(A)** Crystal structure of MNK-2 complexed with staurosporine (PDB: 2HW7); the atoms of the substructure used in the study of pharmacophore fitting screening are labeled in green; **(B)** the lead optimization strategies starting from fragment ET-38766 to clinical candidate ETC-206.

Availability of the crystal structure of MNK2 complexed with staurosporine (PDB: 2HW7) enabled creating minimal substructure, required for crucial interactions with MNK2 (Figure 8). The GOLD software (Jones et al., 1997) used in this study was able to reproduce the binding mode of staurosporine in MNK2. The virtual substructure was docked in the crystal structure of MNK1 (PDB: 2HW6) to similarly elucidate crucial amino acid interactions in the ATP binding pocket. MNK1 pharmacophore prepared in this way was used for pharmacophore fitting study, as a molecular docking alternative and 92 compounds were extracted according to their goodness of fit with the pre-defined substructure.

Structure-based virtual screening protocols were combined with ligand-based virtual screenings of CNIO database and external virtual database of compounds collected by the authors who performed this study. These strategies involved 2D-substructural searches, 2D Tanimoto structural similarity, Feature Trees similarity, and three-dimensional shape and electrostatic similarities based on two reported MNK1 inhibitors. Finally, the authors selected 1,236 compounds for biochemical MNK1 assay and 26 of them were active. The hit ratio of this screening was 2.10% and 10 different scaffolds were represented. Interestingly, one compound (Figure 8, compound 29) demonstrated nanomolar MNK1 (IC_50_ = 646 nM) and MNK2 (IC_50_ = 575 nM) inhibition. Additionally, at the cellular level, compound 29 showed an antiproliferative effect against acute myeloid leukemia cell line (MV4:11, EC_50_ = 17 μM) with dose-dependent decrease in phosphorylation on serine 209 in eIF4E. In conclusion, this study identified 26 hit molecules as MNK1 inhibitors, with 19 of them as fragments with high ligand efficiency values. Among the 26 identified hits, there were 10 diverse chemotypes represented for further drug design studies.

Researchers from A-STAR were particularly interested in imidazopyridazine scaffold (chemotype III in the study of Oyarzabal et al.) as a starting fragment for lead optimization of MNK1/2 inhibitors (Yang et al., 2018). Extensive SAR study of imidazopyridazine derivatives was based on *in silico* conclusions defined in their previous computational study (Kannan et al., 2017). Concisely, researchers in this study aimed to modify the heterocyclic core in positions 3 and 6, with later modification of the imidazopyridazine scaffold (swapping cores strategy). All the derivatives synthesized in this study were firstly examined by molecular docking studies in Glide 2017-3 software (www.schrodinger.com). By detailed computational analysis of the important amino acid residues in the ATP pocket of MNK1/2 kinases, researchers performed initial lead optimization of the fragment (compound ET-38766) to compound 27 (Figure 8B). Novel compound 27 bears imidazopyrazine scaffold, with improved potency against MNK-1 and MNK-2, cell permeability and improved pharmacokinetic properties. After finding optimal substituents in positions 3 and 6, the final step of lead optimization was focused on detailed DFT study to select the final heterocyclic core of MNK1/2 inhibitors. Initially, it was unclear from molecular dynamics (MD) simulations whether the imidazopyrazine N-7 contributes favorably to the binding affinity of MNK inhibitors. To examine this, the authors performed DFT study and demonstrated that N-7 is mostly solvent exposed, thus the final selected heterocyclic core was imidazopyridine. The most promising compound 48 (Figure 8B) later designated as ETC-206, was presented as superior compared to other derivatives in the study. This compound was investigated for the synergism with dasatinib *in vivo* and currently is in phase I clinical trial for the blast crisis chronic myeloid leukemia (BC-CML).

##### Computational approaches in rational discovery of allosteric kinase inhibitors

Although targeting of highly conserved ATP-binding site by Type I and Type II inhibitors provides limited selectivity, inhibiting multiple kinases with a single small-molecule inhibitor was proven to be a useful strategy for therapeutic intervention. However, development of highly selective small-molecule kinase inhibitors remains a pressing concern where targeting of allosteric sites emerged as a promising approach (Wu et al., 2015a). Some of the advantages of targeting allosteric sites include increased selectivity and low toxicity of such inhibitors due to low evolutional conservation of allosteric sites compared to orthosteric (ATP-binding) sites (Fang et al., 2013). Additionally, overcoming of point mutation-associated drug resistance, especially for mutations in the ATP-binding site reported for almost all of ATP-competitive inhibitors, could be another advantage of developing allosteric kinase inhibitors (Gibbons et al., 2012).

While exploitation of allosteric sites represents a very promising strategy, it remains challenging from the aspect of rational drug discovery. Some of the major obstacles include identification of allosteric binding sites, which are usually hidden in less populated higher energy conformations of the proteins. Those conformations are poorly accessible to current experimental methods of structural biology (Lu et al., 2018a). Additionally, allosteric effectors are susceptible to “mode switching,” where minor chemical modification of ligand induces critical change in activity (Wenthur et al., 2014). Although known CADD workflows for discovery of drugs directed to orthosteric binding sites are being used in allosteric inhibitors discovery (Rastelli et al., 2014; Schoepfer et al., 2018), they provide limited utility rising the need for development of more spatialized tools and workflows (Greener and Sternberg, 2018).

Identification of allosteric pockets is a crucial first step in rational discovery of allosteric inhibitors. As will discussed below, a plethora of computationally inexpensive methodologies have been developed for this purpose and many of them are even implemented as web servers. While these methodologies provide fast and inexpensive highway in the discovery of druggable allosteric pockets, proper understanding of the allosteric mechanism is impossible without considering underlying conformational landscape and free-energy profiles where more computationally demanding molecular dynamics based approaches have a predominant role. In this review, we discuss few examples of computational methodologies used for direct discovery of novel allosteric sites and/or allosteric kinase inhibitors. For detailed description of recent breakthroughs in computational methodologies used for allosteric inhibitors discovery in general, the interested reader is referred to the recent reviews (Wagner et al., 2016; Lu et al., 2019).

#### Automatic Computational Tools/Web Servers to Investigate Allostery

Structure-based computational tools AlloSite and recently advanced descendant AllositePro (http://mdl.shsmu.edu.cn/AST/) are intended for fast detection of allosteric site in input PDB structures. Initial detection of allosteric sites is based on Fpocket, a fast open source protein pocket detection software package based on Voronoi tessellation (Le Guilloux et al., 2009). While Allosite uses a machine-learning model to re-rank detected pockets in terms of their allosteric character, AllositePro additionally implements normal-mode analysis (NMA) perturbation with elastic network models to account for protein flexibility. NMA is a technique developed for investigation of the vibrational motion of a harmonic oscillating system in the immediate vicinity of its equilibrium. Under assumption that the potential energy landscape in the vicinity of a minimized atomic structure is approximately harmonic, NMA eliminates the need to integrate the equations of motion and makes NMA much less computationally demanding compared to MD (Bahar and Rader, 2005). Zhang et al. demonstrated utility of AllositePro in identification of novel allosteric site on CDK2 kinase. Existence of novel site was validated in mutagenic analysis (Song et al., 2017). Recently, the same group developed AlloFinder, integrated allosterome mapping, and virtual screening workflow implemented as web server (http://mdl.shsmu.edu.cn/ALF/). AlloFinder relies on AllositePro algorithm for detection of allosteric sites, Allolike filter for pre-filtering of ligand library to enrich allosteric-like compounds (Wang et al., 2012), AutoDock Vina algorithm for docking (Trott and Olson, 2010), and Alloscore empirical scoring function for scoring allosteric modulator-protein complexes (Li et al., 2016). In the final step, alosterome mapping is used to detect highly similar allosteric sites among known human allosteric sites and to rule out selective ligands. This approach was retrospectively validated on several kinase targets (Huang et al., 2018).

CavityPlus (http://www.pkumdl.cn:8000/cavityplus/index.php) is another web server for detection of potential allosteric sites that works on similar principle (Xu et al., 2018). CavityPlus is aimed to detect potential binding sites on the surface of a given protein and rank them based on ligandability and druggability scores. This server integrates several functionalities: CAVITY for detection and scoring of potential binding sites (Yuan et al., 2013); CavPharmer for generation of receptor-based pharmacophores (Chen et al., 2014); CorrSite for prediction of allostery based on NMA motion correlation analysis between allosteric and orthosteric sites (Ma et al., 2016); CivCys for detection of binding sites for covalent inhibitors (Zhang et al., 2017). Functionalities of CavityPlus were successfully used for identification of allosteric binding site on Polo-like kinase 1 (Plk1). Subsequent molecular-docking-based virtual screening on allosteric site resulted in identification of few potent Plk1 inhibitors (Yun et al., 2016).

Another successful implementation of web server based tools for allosteric drug discovery is Kinase Atlas (https://kinase-atlas.bu.edu/) (Yueh et al., 2019). Kinase Atlas is systematic collection of mostly unexplored allosteric sites (binding hot spots) calculated for 4,910 PDB structures of 376 distinct kinases. The hot spots are identified by FTMap. This method places molecular probes (small organic molecules) on a dense grid around the protein and finds favorable positions using an empirical energy function and CHARMM potential. After clustering of obtained positions for each probe, regions that bind several probe clusters are marked as hot spots (Kozakov et al., 2015). Authors of the study identified novel allosteric site on CDK2 and screened library of 1,280 molecules using disulphide-based fragment screening. Two potent and novel allosteric inhibitors were described.

#### Molecular Dynamics-Based Approaches to Investigate Allostery

Molecular dynamics-based approaches in rational discovery of allosteric kinase inhibitors have potential to provide exclusive insight in atomic-level dynamical mechanism of allostery, to explore conformational landscape and capture kinase conformational states inaccessible to current experimental methodologies. Therefore, molecular-dynamics-based approaches, even though being computationally intensive, could detect previously unknown conformations and hidden allosteric binding pockets (Guo and Zhou, 2016; Lu et al., 2018a).

Combination of conventional MD simulations with other standard SBDD approaches resulted in identification of novel allosteric sites and discovery of novel allosteric ligands in several cases. For example, Perez et al. identified novel inhibitory allosteric site and inhibitors of p38α by using MD simulations starting from the X-ray structure of binary complex of p38α and its interacting partner MAPK-activated protein kinase 2 (MK2). MD simulations permitted definition of pharmacophoric features of small peptide inhibitors derived from sequence of MK2. Subsequent virtual screening study resulted in first small molecule allosteric inhibitor for identified binding site (Gomez-Gutierrez et al., 2016). Cournia et al. verified existence of allosteric site on human PI3Kα previously described in murine PI3Kα using combination of FTMap, MD, and *in vitro* assays. Intriguingly, MD simulations revealed different binding mode of studied allosteric inhibitor in murine, WT, and mutant forms of PI3Kα and consequent differences in propagation of allosteric signal to orthosteric ATP-binding site (Gkeka et al., 2015).

Computational costs of insufficient conformational sampling often limit application of conventional MD simulations in investigating allostery phenomena. Currently, there is a large gap between the time scale which can be reached in MD simulations and that observed in experiments. Several strategies for enhancing the sampling of MD simulations have been proposed (Aci-Seche et al., 2016; Yang et al., 2019). Two recently reported studies demonstrating full power of enhanced sampling methods (Markov-state modeling based adaptive sampling and parallel tempering in the well-tempered ensemble) are discussed below with special reference to atomic-level description of allosteric communication and discovery of cryptic allosteric pockets.

Pande et al. investigated activation pathway of c-Src kinase using massively distributed MD simulations (550 μs) on Folding@HOME (Shirts and Pande, 2000) Markov-state modeling (MSM) and adaptive sampling algorithms in order to provide description of factors underlying thermodynamics and kinetics of c-Src activation and to identify key structural intermediates (Shukla et al., 2014). Briefly, MSM models represent kinetical description of a system's underlying free-energy landscape, useful for characterization of probability of dynamical transitions between conformational states identified in many independent MD simulations and for extrapolation of long time system's behavior (Sengupta and Strodel, 2018). In this study intermediate conformational state which could be stabilized to block the c-Src activation pathway, was described through MSM analysis for the first time. Further analysis on identified c-Src conformational state revealed the existence of allosteric pocket and surprisingly high structural similarity to known complex of CDK2 bound to allosteric inhibitor—ANS (Betzi et al., 2011). Further simulations confirmed binding of ANS to the novel allosteric site of c-Src and blockage of activation process by stabilization of intermediate states. Additionally, the long-range residues coupling analysis identified myristate-binding pocket as another potential target for development of allosteric modulators of c-Src. Taken together, results of this study highlighted large-scale MD coupled with MSM modeling as an indispensable tool for identification of novel conformational states, potential allosteric pockets, and study of mechanisms of allostery in kinases.

In another example, authors explored the possibility of bidirectional communication between allosteric so-called PIF-pocket and ATP-binding site in PDK1 protein kinase using a combination of experimental techniques and enhanced-sampling simulations [parallel tempering simulations in the well-tempered ensemble (PT-WTE)] (Schulze et al., 2016). Results of PT-WTE MD revealed bidirectional mechanisms of communication between the ATP-binding site and allosteric site. Interestingly, this study for the first time demonstrated how different ligands which bind to the ATP-binding site differently modulate responses of allosteric site in interaction with a partner protein (e.g., enhance or inhibit interaction). Providing computer platform for rational design of allosteric modulators, the authors of this study opened an exciting avenue for future discovery of novel class of kinase inhibitors with less on-target side effects and more specific modulation of signaling pathways.

#### Case Study

##### Rational design of clinical candidate Asciminib—allosteric Bcr-Abl1 inhibitor

**Figure 9:**
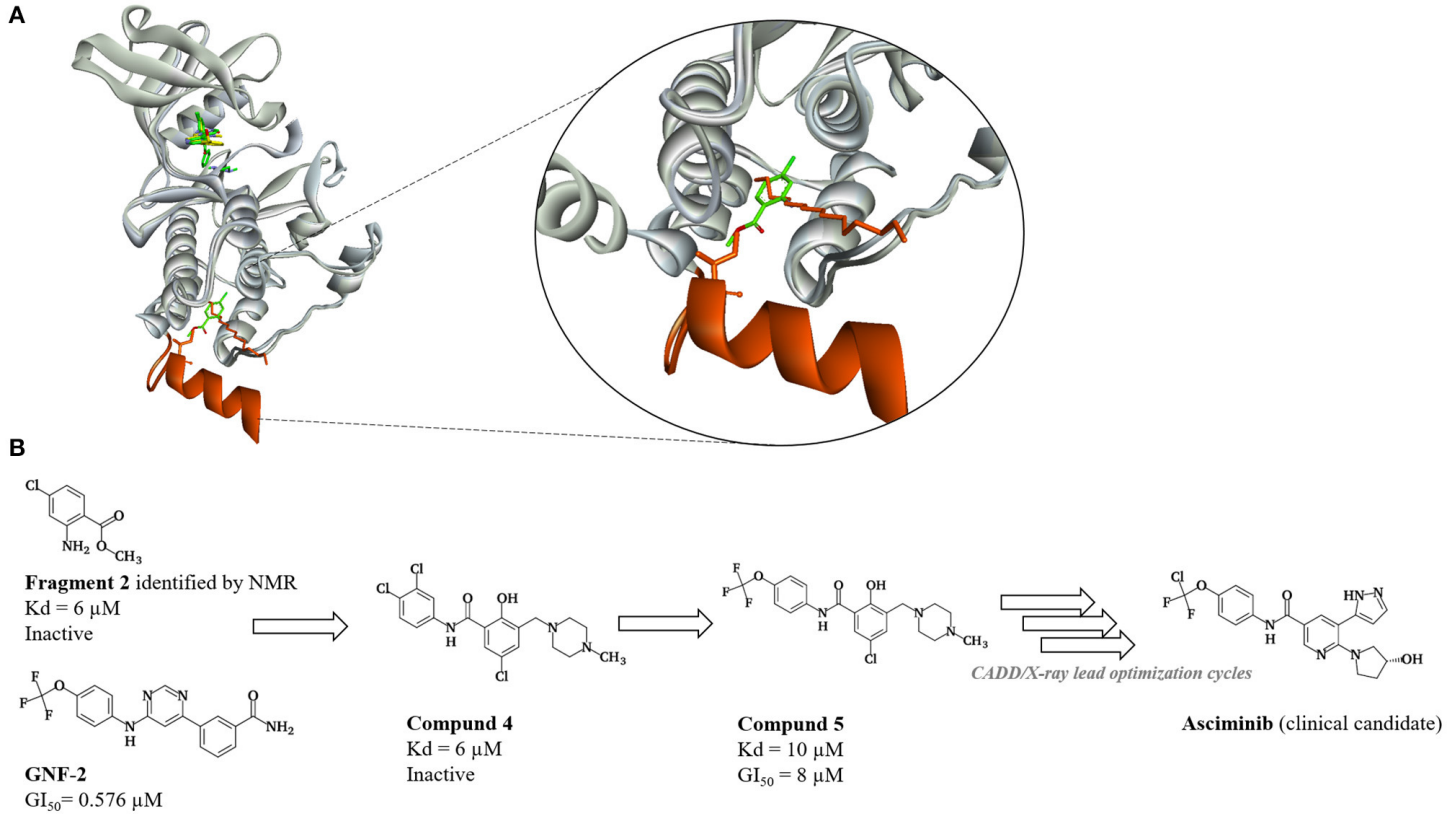
Discovery of Asciminib (Schoepfer et al., 2018). **(A)** Representation of initial hypothesis regarding bending of helix I (orange). Assembled inactive state of ABL1 kinase in complex with myristic acid (orange sticks) (PDB: 1OPL, SH3, and SH2 domains omitted for clarity) is superimposed on ABL1 in complex with fragment 2 (green sticks) (PDB: 3MS9). Steric clash between isoleucin I521 (orange sticks) on helix I and fragment 2 prevents full bending of helix I and formation of assembled inactive state of ABL1. Helix I is not visible in the PDB: 3MS9. **(B)** Medicinal chemistry progression from fragment 2 to fragment derived hit 4, first active hit–compound 5 and finally clinical candidate—Asciminib.

Asciminib belongs to a class of drugs designed to inhibit Bcr-Abl by binding to an allosteric pocket known as myristate-binding pocket. Rational development of Asciminib started with fragment-based screening using NMR assay (Schoepfer et al., 2018). Although determined NMR-based dissociation constants (Kd) for fragment hits were satisfactory, none of the fragments were active in biochemical and cellular assays. Subsequent X-ray studies revealed inability of fragment hits to induce assembled inactive state by bending of helix I, previously reported as conformational change important or autoinhibition of Abl by myristoilation (Nagar et al., 2003). Following this finding, the authors established another screening assay, the NMR-based conformational assay, which monitors the conformational state of C-terminal helix I (Jahnke et al., 2010). NMR-based conformational assay was used to investigate identified fragments and series of known allosteric modulators—derivatives of GNF-2 (Adrian et al., 2006; Figure 9). Results of the study revealed that compounds which bind to myristoyl pocket and do not induce helix I bending were actually functional activators of Abl1 (by interfering with autoinhibition mechanism of Abl1). Critical bending of helix I was found to be induced by the presence of CF_3_O– group from GNF-2. Based on these findings, CADD techniques (molecular docking, similarity and pharmacophore searches) were used to design compound **X** in respect to X-ray structure with bent helix I conformation. Subsequent introduction of CF_3_O– group finally led to the first active allosteric inhibitor. Molecular modeling techniques were used in combination with X-ray crystallography in order to optimize potency and drug-like properties of the compound. Although only standard CADD techniques were reported in the discovery of Asciminib, recent application of molecular dynamics-based approaches demonstrated utility of such techniques in examination of mechanisms of resistance and effects of dual targeting of ATP-binding and allosteric site providing rationale for development of novel drugs (El Rashedy et al., 2018; Meng et al., 2018; Zhan et al., 2019).

##### Machine learning methods to predict kinase-compound interactions

Nowadays, we are seeing the widespread use of machine learning in many areas, including pharmaceutical industry, especially in drug design. Popular computational methods initially used in pharmaceutical research were quantitative structure activity relationships (QSAR) and quantitative structure property relationship (QSPR), which were adequate for small datasets. However, with the rapid growth of databases (thanks to methods such as high-throughput *in vitro* screening and X-ray crystallography), it became inevitable to develop different *in silico* tools that can manage bigger data (Ekins, 2016). Today, many different machine learning methods such as support vector machines (SVM), k-Nearest Neighbors, Artificial Neural Networks (ANN), Deep Learning (DL), *etc*. are used in pharmaceutical research and they can be applied in various processes of drug design from virtual screening to *de novo* drug design (Buchwald et al., 2011; Drewry et al., 2017; Konze et al., 2019; Kuthuru et al., 2019; Lee et al., 2019; Zhavoronkov et al., 2019).

Many different machine learning models were created for the prediction of drug–target interactions (DTI), and many DTI methods have been applied to the protein kinases family (Kuthuru et al., 2019). Unlike LB and SB methods, DTI prediction uses the information from both protein and ligand and these methods can be similarity based or descriptor-based. One of the first similarity-based methods for identification of drug–target interactions was introduced by Yamanishi et al. in 2008. It used the known drug structure, protein sequence and drug–target interaction network to determine unknown ligand–target interactions. The main hypothesis is that two compounds that have high structure similarity might probably interact with similar target proteins, and *vice versa* two proteins with high sequence similarity might probably interact with similar drugs (Yamanishi et al., 2008). On the other hand, descriptor-based models use feature vectors from known drug structures and protein sequences, as inputs for machine learning methods, such SVM, AAN, DL, etc. In 2011, Buchwald et al. used SVM to prepare the model for prediction of protein kinases–ligand interactions. They used a set of binding data obtained from 113 different protein kinases and 20 inhibitors obtained through ATP site-dependent binding competition assays. They focused on vector features that describe the structure of molecules that are connected with certain chemical environment–protein active site sequence and created a SVM model with good predictivity (Buchwald et al., 2011).

Recently, the use of ANN, especially deep learning methods saw a significant increase in the process of drug design (Ekins, 2016; Merk et al., 2018; Putin et al., 2018; Konze et al., 2019). Deep generative models are utilizing neural networks to generate new objects (drugs) with desired properties (for example activity, Ki, IC_50_). These methods should be able to produce chemically correct structures without the need for including fragment libraries and/or rules for their combination (Merk et al., 2018). The ability to produce novel chemical structures with certain properties makes deep generative models suitable for the discovery of novel possible therapeutics (Zhavoronkov et al., 2019). In 2018, Merk et al. applied generative models to come up with novel bioactive, synthesizable drugs. They trained the model with more than 500,000 SMILES of bioactive compounds with their activity properties extracted from the ChEMBL (K_D_, K_i_, IC/EC_50_ values <1 μM). Additionally, the model was fine-tuned to enable the *de novo* generation of target-specific ligands on retinoid X receptors (RXR) and/or peroxisome proliferator-activated receptors (PPAR). Finally, none of the generated compounds was identical to compounds from the training sets, and they were residing within the RXR/PPAR region of the fine-tuning set (Merk et al., 2018).

#### Case Studies

##### Predictive proteochemometric models for kinases derived from 3D protein field-based descriptors

Subramanian et al. described the development of proteochemometric models for 1,572 inhibitors and 95 kinases obtained from Kinase SARfari (https://chembl.gitbook.io/chembl-interface-documentation/legacy-resources#kinase-sarfari) and CHEMBL database, using 3D structure of proteins and active and inactive ligands. Proteins were described with molecular interaction fields derived from Schrödinger's WaterMaps, while different 1D, 2D, and 3D descriptors were used to describe the ligands. Separate training sets were created for the ligands and targets. Different methods were used for preparation of the proteochemometric models: support vector machines (SVM) and random forests (RF). The ligand prediction model was trained on the ligand training set and was used for ligand prediction model and target training set for target predicting model. In the end, they validated all the models using internal and external validation. This approach allows creation of not only predictive proteochemometrics model for protein kinases, but also preparation of visually interpretable models. This allows interpretation of kinase–ligand interactions, which can be used, for example, for optimization of ligand in order to achieve optimal activity and/or selectivity. Having visually interpretable models is the advantage compared to classical DTI methods that use only 2D information (Subramanian et al., 2013, 2016).

##### Deep learning model for identification of potent discoidin domain receptor 1 kinase inhibitors

Recently, Zhavoronkov et al. created a deep generative model for *de novo* small-molecule design—GENTRL (GENerative Tensorial Reinforcement Learning). Besides the effectiveness of a compound against a given biological target, GENTRL also takes into account its dissimilarity from other molecules in the literature and patent space, as well as its synthetic feasibility. For the proof-of-concept GENTRL was used to design potential Discoidin domain receptor 1 (DDR1) kinase inhibitors. Data was collected from different data sets: ZINC data set, known DDR1 kinase inhibitors data set, common kinase inhibitors, molecules with activity on non-kinase targets, patent data, and used to train the model. The model was generated by combining reinforcement learning with a reward, variational inference, and tensor decompositions. Finally the randomly elected six compounds that have not been previously published or patented were designed, synthesized, and experimentally tested. The whole process lasted only 46 days, which suggests that the application of drug design methods such as this will reduce the time and cost of drug discovery process (Zhavoronkov et al., 2019).

## Concluding Remarks

*In silico* approaches are viable, usually cheaper and faster alternative to experimental drug discovery techniques. This review summarizes the most important computational tools that have led to the discovery of kinase inhibitors, many of which are in clinical use today as promising anticancer drugs. Computational approaches, such as QSAR modeling, ligand-based and structure-based virtual screening, molecular docking, molecular dynamics, quantum mechanics, fragment-based drug design, and machine learning methods, provide unique insight in the conformational landscape of kinases, structural requirements for inhibitory activity, binding modes and atomistic mechanisms of allostery, which represent indispensable information for rational *de novo* design. One of the main advantages of computational approaches is the possibility of introduction of new groups on the known scaffolds and *in silico* prediction of activities and binding affinities. Known scaffolds of the approved KIs include pyrimidine (imatinib, dasatinib, nilotinib), quinazoline (erlotinib, gefitinib, afatinib, vandetanib), pyridine (sorafenib), pirrolopyridine (vemurafenib), pyrazolopyridine (ibrutinib) etc. *In silico* modification of these scaffolds resulted in the design of many kinase inhibitors with enhanced predicted activities and binding affinities which can serve as lead compounds for further synthesis and preclinical testing. New chemical scaffolds that possess kinase inhibitory activity (imidazopyridazine, imidazopyridine, isoquinoline, phenazinamine, etc.) have also been proposed by computational approach and represent a good starting point for discovery of new kinase inhibitors. Due to increases in computational power, algorithmic improvements and increased accuracy, *in silico* approaches are yet expected to radically shape the era of kinase inhibitor discovery. Of note is to emphasize that not all drug discovery projects could be initiated and guided only with computational studies. The computational chemist must be aware of the structural biology of the studied targets, their dynamical changes influenced upon fragment/ligand binding. Whenever possible, it is advised to start CADD studies with experimental data and continue *in silico* optimization with combined modeling approaches, as much as possible. This review highlights the recent advances in discovery of kinase inhibitors by *in silico* approaches and can be useful for future design and synthesis of new kinase inhibitors as anticancer drugs.

## Author Contributions

ZG wrote the introduction and LB methods. TD wrote the structure of protein kinases, machine learning methods, and SB methods. DR wrote FB methods. ND wrote modeling of allosteric kinase inhibitors. KN contributed to the conception of the manuscript, collection of the data, drafting and revising. All authors have contributed to the bibliographical research and interpretation of the work, to its critical revision, and approved the final version of the manuscript.

### Conflict of Interest

The authors declare that the research was conducted in the absence of any commercial or financial relationships that could be construed as a potential conflict of interest.
